# Divergent and convergent modes of interaction between wheat and *Puccinia graminis* f. sp*. tritici* isolates revealed by the comparative gene co-expression network and genome analyses

**DOI:** 10.1186/s12864-017-3678-6

**Published:** 2017-04-12

**Authors:** William B. Rutter, Andres Salcedo, Alina Akhunova, Fei He, Shichen Wang, Hanquan Liang, Robert L. Bowden, Eduard Akhunov

**Affiliations:** 1 Department of Plant Pathology, Kansas State University, Manhattan, KS 66506 USA; 2 Integrated Genomics Facility, Kansas State University, Manhattan, KS 66506 USA; 3 USDA ARS, Hard Winter Wheat Genetics Research Unit, Throckmorton Plant Sciences Center, Manhattan, KS 66506 USA; 4 USDA-ARS, U.S. Vegetable Laboratory, 2700 Savannah Highway, Charleston, SC 29414 USA; 5 TEES-AgriLife Center for Bioinformatics and Genomic Systems Engineering, Texas A&M University, 101 Gateway, Suite A, College Station, TX 77845 USA; 6 School of Data and Computer Science, Sun Yat-sen University, Guangzhou, 510006 China

**Keywords:** Wheat resistance, Stem rust, Comparative genomics, Gene co-expression network

## Abstract

**Background:**

Two opposing evolutionary constraints exert pressure on plant pathogens: one to diversify virulence factors in order to evade plant defenses, and the other to retain virulence factors critical for maintaining a compatible interaction with the plant host. To better understand how the diversified arsenals of fungal genes promote interaction with the same compatible wheat line, we performed a comparative genomic analysis of two North American isolates of *Puccinia graminis f. sp. tritici* (*Pgt*).

**Results:**

The patterns of inter-isolate divergence in the secreted candidate effector genes were compared with the levels of conservation and divergence of plant-pathogen gene co-expression networks (GCN) developed for each isolate. Comprative genomic analyses revealed substantial level of interisolate divergence in effector gene complement and sequence divergence. Gene Ontology (GO) analyses of the conserved and unique parts of the isolate-specific GCNs identified a number of conserved host pathways targeted by both isolates. Interestingly, the degree of inter-isolate sub-network conservation varied widely for the different host pathways and was positively associated with the proportion of conserved effector candidates associated with each sub-network. While different *Pgt* isolates tended to exploit similar wheat pathways for infection, the mode of plant-pathogen interaction varied for different pathways with some pathways being associated with the conserved set of effectors and others being linked with the diverged or isolate-specific effectors.

**Conclusions:**

Our data suggest that at the intra-species level pathogen populations likely maintain divergent sets of effectors capable of targeting the same plant host pathways. This functional redundancy may play an important role in the dynamic of the “arms-race” between host and pathogen serving as the basis for diverse virulence strategies and creating conditions where mutations in certain effector groups will not have a major effect on the pathogen’s ability to infect the host.

**Electronic supplementary material:**

The online version of this article (doi:10.1186/s12864-017-3678-6) contains supplementary material, which is available to authorized users.

## Background

The fungal pathogen *Puccinia graminis f. sp. tritici* (*Pgt*) is the causal agent of wheat stem rust that poses a major threat to wheat production around the world [1–3]. Like other biotrophic plant pathogens, *Pgt* infects susceptible host plants by engaging in a complex interaction that involves multiple proteins from both the fungus and plant. Effector proteins secreted by the fungus interact directly with host plant factors and function to alter plant cellular defenses, architecture, and metabolism, ultimately leading to a compatible plant-pathogen interaction [4–8]. Recognition of effector proteins by plant host resistance genes forms the basis of effector-triggered immunity and drives fast evolution of effector complement in the pathogen populations [9, 10]. Mutations in effector encoding genes are suggested to be one of the major factors rendering resistance genes ineffective against new pathogen populations [11–17]. It has been suggested that the rate of resistance gene breakdown may be accelerated by the modern agricultural practice of planting a limited number of crop genotypes every year over large areas, thereby facilitating quick selection of rare virulent pathogen mutants. The resulting pathogen population shifts were shown to be linked with significant losses in crop production in last decades [2, 18].

The analysis of infected tissue transcriptomes has been shown to be a powerful tool for gathering systems level information about the plant-fungal interaction. Previous studies have shown that infection of hexaploid wheat (*Triticum aestivum)* by biotrophic fungal pathogens results in substantial transcriptional changes in both the host and pathogen [19–24]. Analysis of these transcriptomic datasets has revealed general systems level patterns in the response of *T. aestivum* to invasion by specific species of rust fungi [24]. However, there is currently limited information available on how differences in the effector complements of distinct rust isolates affect the transcriptional response of wheat plants. Considering the importance of effectors in manipulating the host biological pathways and the high rate of effector sequence and content evolution [10, 14, 16], it is reasonable to hypothesize that the distinct effector sets of diverged *Pgt* isolates can alter how they interact with the same susceptible host plant. To better understand how genomic differences amongst rust isolates affect the host’s transcriptional responses we have sequenced two North American isolates of *Pgt* to identify both conserved and polymorphic groups of effectors in each. These isolates were used to infect the same susceptible wheat host, and RNA-seq analysis of infected leaf tissues was performed to generate time-course GCNs for each isolate. The comparative analysis of plant-pathogen GCNs and rust isolate genomes revealed both isolate-specific and conserved wheat pathways modulated during infection. These results provided insights into how different *Pgt* isolates can utilize diverged and conserved set of effectors to modulate specific host plant systems to establish compatible interaction.

## Methods

### Sample collection and sequencing

To generate time-course transcriptome profiles of *Pgt* infected leaf tissues, urediniospores from either RKQQC or MCCFC *Pgt* races were inoculated separately onto 14 day-old wheat seedlings (cv. Morocco). Plants were inoculated using a 1% suspension of urediniospores (v/v) in Soltrol 170 (Philips 66, Bartlesville, OK) that was sprayed onto leaves using an atomizer at 10 psi. Following inoculation, plants were kept for 20 min at room temperature and then incubated in a dew chamber at 100% humidity for 24 h at 18 °C. Plants were then moved back into growth chambers. Inoculated wheat was grown in a controlled growth chamber environment (16 h light, 8 h dark, 22 °C), and three biological replicates were subsequently collected at 6 time points (0, 12, 24, 48, 72, and 96 h after inoculation). Total RNA was extracted using TRIzol reagent (Life Technologies) according to the manufacturer’s guidelines. The quality of RNA samples was assessed with the Agilent 2200 TapeStation using the RNA ScreenTape assays. The sample quantification was performed with the Qubit 2.0 Fluorometer (Thermo Fisher Scientific). Nucleic acid purity (A260/280 and A260/230) and quantity was evaluated using the NanoDrop ND-1000 Spectrophotometer (Thermo Fisher Scientific). The RNA-seq sequencing libraries were constructed using the TruSeq RNA Library Prep Kit (Illumina) according to the manufacturer’s protocol. Each library was validated and quantified using the Bioanalyzer instrument (Agilent). Pooled libraries were sequenced (4 pooled libraries per lane) using HiSeq2500 sequencing instrument (1 × 100 bp run). Raw sequencing reads were processed using Cutadapt v1.4.1 [25] and FASTX-Toolkit (http://hannonlab.cshl.edu/fastx_toolkit/) to remove adapters and low quality bases. The quality trimming and filtering was performed using the following criteria: bases should have minimum quality score of 15 and a minimum length of 30 bp. The low-quality reads with less 80% of their bases having quality scores of at least 15 were removed.

Genomic DNA samples were isolated from the fungal urediniospores collected from the *Pgt* cultures bulked from a single pustule isolation. Each *Pgt* isolate was grown in isolation on a susceptible wheat line (cv. Morocco) in a controlled growth chamber environment (see above) and underwent through three rounds of spore collection and inoculation to develop adequate sample quantity. Once adequate tissue samples were obtained, genomic DNA was extracted from approximately 0.75 g of urediniospores using the OmniPrep fungal DNA extraction kit (G-Biosciences) according to the manufacturer’s protocol. The quality of DNA samples was assessed with the Agilent 2200 TapeStation using the Genomic DNA Analysis Tapes. DNA sample quantification was performed with the Qubit 2.0 Fluorometer (Thermo Fisher Scientific). Nucleic acid purity (A260/280 and A260/230) and quantity was evaluated using the NanoDrop ND-1000 spectrophotometer (Thermo Fisher Scientific). Paired-end sequencing libraries were constructed using the NEBNext DNA library prep kit (New England Biolabs) according to the manufacturer’s instructions. Libraries were sequenced at the Kansas State Integrated Genomics Facility (IGF) on the MiSeq instrument using the 600 cycles MiSeq reagent. Pacific Bioscience reads were generated using 1 SMRT cell of PacBio RS II using P6C4 PacBio chemistry at UC Davis Genome Center (see Additional file 1: Table S1 for sequencing data summaries).

### Fungal genomic data analyses

Paired-end Illumina reads generated from the fungal genomic DNA libraries were trimmed for adapters using Cutadapt v1.4.1, remaining bases were trimmed to a quality score of 15, and filtered to a minimum length of 30 bp using FASTX-Toolkit (http://hannonlab.cshl.edu/fastx_toolkit/). Low quality reads were discarded. Paired-end reads from both MCCFC and RKQQC *Pgt* isolates were aligned against the publically available SCCL reference sequence (http://www.broadinstitute.org/) using bowtie2 v2.2.1 [26] using the default settings. The summary statistics for each alignment were assessed using the Picard tools (https://broadinstitute.github.io/picard/). Resulting alignment files were used as inputs for the GATK UnifiedGenotyper [27] to call variants using the default settings. Multiallelic sites with more than 2 alleles were discarded. The resulting VCF file was filtered using vcftools (v 0.1.12b) [28] with the filter parameters set requiring a minimum overall read coverage depth of 15× across both isolates with a non-reference allele frequency of 0.3. To reduce false variant calling rate in the highly duplicated genomic regions, a maximum read coverage depth for each variable site was set to be two times the mean coverage depth for entire genome. The filtered VCF files were used as input for SNPeffect [29] to predict the functional effect of each DNA sequence variant.

*De novo* genome assembly of genomic reads from individual isolates was performed using CLC bio assembly software (QIAGEN). The resulting contigs were further extended by incorporating long PacBio reads using PBSuite v14.9.9. The contigs that were shorter than 300 bp were removed from the assemblies. To remove possible sample contamination from entophytic microbes and other non-fungal sources, all assembled contigs were compared against the NCBI non-redundant nucleotide database using the BLASTN tool. The best BLASTN hits were used to determine the likely taxa of origin for each contig. Only contigs with the top BLASTN hits to fungal sequences were retained. Assembly quality statistics for each genome were generated using the QUAST software (v4.0) [30]. The assessment of the completeness of genome assembly using the single copy orthologous genes was performed using the BUSCO software in the genome analysis mode [31].

### De novo transcript assembly, genome annotation, and gene diversity analysis

RNA-seq reads were aligned to the wheat genomic reference [32]. All unmapped reads from both isolates were used to create *de novo* transcript assembly with Trinityrnaseq v2.0.6 program [33]. The *de novo* assembled transcript assemblies were combined with the publically available SCCL transcripts, and used to separately annotate gene models within the genome assemblies from each isolate using the PASA annotation software. The proteins predicted in four fungal genomes (*Pgt* races SCCL, MCCFC, and RKQQC, and *Puccinia triticinia*) were clustered using OrthoMCL v2.0.9 [34].

To predict effector candidates, N-terminal signal peptides and transmembrane domains were predicted using SignalP v4.1 and TMHMM v2.0 software with default settings [35]. A protein was considered an effector candidate only if it contained a predicted N-terminal signal peptide and lacked a transmembrane outside the first 60 amino-acids of it’s sequence. Using custom R scripts that integrated gene expression, SNP annotation and comparative genome analysis data the genes encoding effector candidates were classified into one of the three categories: present/absent, conserved or divergent. Briefly, genes were considered to be present in a specific *Pgt* isolate genome if they were expressed (FPKM > 0) and they had an annotated gene model. If genes were not expressed or had no an annotated gene model in the respective *Pgt* isolate’s genome assembly, they were considered absent. Those genes found to be present in both isolates were further divided into the groups that were perfectly conserved and contained no identified non-synonymous mutations, and those that were divergent and carried non-synonymous mutations.

### RNA-sequence transcript quantification and network generation

Quality filtered RNA-seq reads were mapped to publically available *Pgt* and the wheat genomes using Tophat (v2.0.10) [36]. To assure that the wheat reads were aligned to the correct homeologous wheat chromosome Tophat parameters were optimized using a subset of reads (see Additional file 1: Figure S1). The parameter optimization achieved a misalignment rate of 0.2% and an overall alignment rate of approximately 75% reads by setting the maximum read segment mismatch equal to 1 using the defaults setting for remaining parameters. The *Pgt* reads were aligned using the default Tophat parameters and achieved an overall alignment rate that varied from 0.2 to 6.8% of the total reads depending on the time point after infection the sample was derived from. For obtaining the expression values of wheat and *Pgt* genes, we used publicly available gene annotations. Aligned reads from both the fungal isolates and wheat were analyzed separately using the *tuxedo* pipeline as described in the Cufflinks2 manual [37]. Briefly, mapped reads from Tophat were assembled and quantified using Cufflinks. A final transcript assembly was generated using the *cuffmerge*, and *cuffdiff* was used to identify differentially expressed genes using the normalized expression estimates in the form of FPKM values. At each of the six time points, we compared gene expression between the RKQQC and MCCFC treatments, as well as the expression of all genes across all tissue sampling time points. To extract and analyze the *cuffdiff* results we used the R package CummeRbund. Only those genes that had an FPKM ≥ 1 in six or more biological replicates, a log2-fold change ≥ 2, and an FDR ≤ 0.05 were used for further expression analysis.

Expression profiles from both fungal and wheat genes were clustered for each isolate treatment separately. Clustering was performed on each RNA-seq dataset by first calculating euclidean distance between each gene using the *agnes* function from the R package ‘*cluster’* to the log2 FPKM +1 transformed and mean centered data. The number of clusters K = 40 was chosen by testing K values ranging from 10 to 500, and selecting the value that locally minimized both the within/between sum of squares value and the CH value as calculated by the summary function from the R package ‘*cluster’*.

Clusters containing at least 10 genes in either wheat or *Pgt* datasets were selected for functional enrichment analysis. The GO annotations for the wheat and *Pgt* genes were retrieved from the Ensemble database. All GO terms were mapped to their parent nodes following the Gene Ontology structure, and ignoring GO terms that are associated with more than 25% of the genes in the genome. The GO term enrichment was tested by the Fisher’s Exact test followed by the Benjamini-Hochberg multiple testing correction. For each cluster, GO terms with corrected *p*-value < 0.05 were defined as significantly enriched. The log2-fold change between a cluster and the genomic background was used to show the level of enrichment.

The GCNs were generated separately for each *Pgt* isolate’s dataset using the host and pathogen genes that showed differential expression either between *Pgt* isolate treatments or across time points over the course of infection. The R package geneNet was used to account for the non-scale free nature of the time-course data [38]. Partial correlations (edges) between the genes (nodes) identified by the algorithm implemented in geneNet were filtered using the FDR ≤ 0.001 threshold. The significant edges (FDR ≤ 0.001) were classified into three groups based on their conservation status between the two isolate GCNs: conserved edges, MCCFC-unique edges, and RKQQC-unique edges. The lists of the wheat genes contained within each edge groups were extracted and annotated using the Blast2GO software with the default settings. Significantly enriched GO terms were identified using the Fisher exact test (FDR < 0.05). The sub-networks of connected genes were extracted for each significantly enriched GO term. The GO term-specific sub-network graphs were generated using the R igraph package.

### Quantitative RT-PCR analysis ABA- and SA-treated leaf tissues

Wheat seedlings (cv. Morocco) were grown in a controlled growth chamber environment (16-h light, 8-h dark, 22 °C) for fourteen days until the 2-leaf development stage. Three biological replicates were generated for each of four foliar applications: mock (aqueous solution alone), abscisic acid (2 mM), and salicylic acid (50 mM and 100 mM). Treatments were all applied as foliar sprays in an aqueous solution (40% Ethanol, 0.05% TritonX-100 + treatment) until leaves were dripping. About 100 mg of leaf tissue was collected from each application 18 h after treatment. Total RNA was extracted using Trizol reagent (Life Technologies) according to manufacturer’s protocol. Each RNA sample was treated with DNase I (Life Technologies) according to manufacturer’s protocols. DNase-treated RNA from each biological replicate was used to generate cDNA for each sample.

Samples from each biological replicate were tested by mixing four technical replicates of the same qPCR reaction mixture using the IQ SYBR green super mix reaction mixture (BIO-Rad) with one set of experimental primers specific for POX2 (5′- GTCATACTGCAGCCTGTTGCCTTC-3′, 5′- GCTTCCCAACTCTACCTAGCTGGATAC-3′), MYBa (5′- GGTGATGGCAGCAGAGGG-3′, 5′- GGCGAGCAGGAACTTCATGGTG-3′), MYBb (5′- CATGAGCCCACTTGGAATGCTAGATAG-3′, 5′- GAGGCAGGCTGGAAGATGGATGAG-3′), and MYBd (5′- GGTGATGGTAGCAGAGGACCAGAG-3′, 5′-ATTCAGCCACAGACGCCATCG-3′) or the house keeping actin primer set (5′- ACCTTCAGTTGCCCAGCAA-3′, 5′- CAGAGTCGAGCACAATACCAGTTG-3′). Each experimental primer set was paired with four technical qPCR replicates of the actin primer. Reactions were carried out on a CFX96 Real-Time system (Bio-Rad) with the same thermal cycler protocol consisting of a one-time 95 °C − 3 min, followed by 40 cycles 95 °C − 15 sec, 60 °C − 30 sec, 72 °C − 40 sec, followed by automatic dissociation analysis to asses primer specificity. Raw fluorescent quantification results for each cycle were used for normalization and to calculate relative transcript abundance using the online Real Time PCR Miner v. 4.0 [39].

## Results

### Comparative genomic analysis of Pgt isolates MCCFC and RKQQC

Genomic sequence similarity was assessed between the two North American isolates of *Pgt* 59KS19 (henceforth, referred to by its race designation MCCFC), 99KS76A-1 (henceforth, referred to by its race designation RKQQC) [40], and the previously sequenced and annotated isolate 75-36-700-3 (henceforth, referred to by its race designation SCCL) [41]. Genomic DNA was extracted from the MCCFC and RKQQC urediniospores and sequenced using both short (2 × 100 bp Illumina reads) and long read (Pacific Biosciences) technologies (Additional file 1: Table S1). In total, 68.3 and 62.8 million quality filtered Illumina paired-end (PE) reads were generated for MCCFC and RKQQC, respectively, and were aligned to the SCCL genomic reference. A total of 50.1 million genomic reads (73.3%) generated from RKQQC, and 25.8 million genomic reads (41.0%) generated from MCCFC were mapped to the SCCL genome. The RKQQC reads covered 84.5% of the SCCL genome at a mean coverage of 81.72×, and the MCCFC genomic reads covered 75.8% of the reference genome at a mean coverage depth of 49.57× (Table 1).

**Table 1:** Summary of the genomic reads from RKQQC and MCCFC aligned to the SCCL reference genome

*Pgt* race	RKQQC	MCCFC
Total reads	62,781,618	68,290,518
Aligned reads	46,024,317	28,014,613
Proportion reads mapped	73.31%	41.02%
Mean coverage depth	81.72	49.56
Median coverage depth	78	49
% of genome with no coverage	15.52%	24.24%
% of genome with 5× coverage	77.96%	73.80%
% of genome with ≥10× coverage	75.07%	70.82%

To characterize the genomic diversity of the newly sequenced *Pgt* isolates, the paired-end genomic reads mapped to the SCCL genome were processed using the variant calling algorithms implemented in the GATK UnifiedGenotyper [27]. A total of 782,717 (read coverage depth ≥ 5 per allele) polymorphic sites including 89,417 indels as well as 693,300 SNPs were identified in the MCCFC and RKQQC genomes. Amongst all the SNPs identified by comparing two isolates, 347,376 (50%) discriminated between MCCFC and RKQQC genotypes, suggesting the high level of inter-isolate genetic divergence (Table 2). The potential functional effects of the identified divergent SNPs were assessed using the publically available SCCL gene models (Table 3). In total, 6,624 (41.92%) of the gene models from the SCCL genome showed the presence of non-synonymous SNPs.

**Table 2:** Summary of variant calls generated using the UnifiedGenotyper from the GATK package

Types of variable sites	Number of sites
Total number of biallelic sites called	782,717
Small insertions/deletions	89,417
SNPs	693,300
Informative SNPs with adequate read coverage in both isolates	557,819
Informative sites with the same genotype call in both isolates	210,443
Discriminatory sites with different genotype call in each isolate	347,376
Discriminatory sites heterozygous in MCCFC	71,264
Discriminatory sites heterozygous in RKQQC	104,945

**Table 3:** Assessment of the tentative functional impact of SNPs using SNPeffect program

SNP origin	Mean/Gene	Median/Gene	Max/Gene	Number Genes	Percentage Total Genes
All SNPs in both isolates					
Non-synonymous mutations	3.11	1	106	8620	0.5455
Synonymous mutations	4.48	1	88	9065	0.5737
No mutations	NA	NA	NA	5526	0.3497
SNPs in RKQQC alone					
Non-synonymous mutations	2.81	1	106	8251	0.5222
Synonymous mutations	4.09	1	84	8738	0.553
No mutations	NA	NA	NA	5850	0.3703
SNPs in MCCFC alone					
Non-synonymous mutations	2.63	0	106	7751	0.4906
Synonymous mutations	3.91	1	88	8196	0.5187
No mutations	NA	NA	NA	6432	0.4071
SNPs differentiating RKQQC from MCCFC					
Non-synonymous mutations	1.83	0	96	6624	0.4192
Synonymous mutations	2.6	0	71	7323	0.4635
No mutations	NA	NA	NA	7247	0.4587

Previous diversity studies of the effector encoding genes showed the elevated proportion of non-synonymous SNPs suggesting that this class of genes is likely subject to directional selection [11]. Consistent with these observations, the proportion of non-synonymous and synonymous SNPs in the effector encoding genes (3,567/4,751) compared to the remaining genes (23,405/37,191) in the *Pgt* genome (Additional file 2: Table S2) in our dataset was significantly different (*χ* ^2^ test = 55.5, p-value = 9.4 × 10^−14^). The mean ratio of non-synonymous to synonymous SNPs in the effector encoding genes (0.98) was 12% higher than that of the remaining genes in the *Pgt* genome (0.86).

To identify the regions of the *Pgt* genome missing in the SCCL reference assembly, *de novo* genome assemblies were produced for each isolate. These assemblies were initially produced with the CLC Bio program (QIAGEN) using the paired-end Illumina reads. The CLC Bio contigs were further extended using the 145,823 and 264,876 PacBio reads generated for MCCFC and RKQQC, respectively. To remove contaminating sequences, the genome assemblies were compared against the NCBI non-redundant nucleotide database, and contigs that showed nucleotide similarity to fungal sequences were retained (see Materials and Methods). The final MCCFC and RKQQC genome assemblies measured 93.3 Mb (N50 7,133 bp) and 107.3 Mb (N50 6,292 bp), respectively (Additional file 1: Table S3), and were comparable with the previously reported *Pgt* genome sizes [16, 41]. The *Pgt* genome assemblies and their annotations can be downloaded from the project website http://129.130.90.211/rustgenomics/Download.

### Pgt isolates show diversity in gene content

To further assess the level of gene conservation between the two sequenced *Pgt* isolates and at the same time obtain gene expression data for host and pathogen, RNA-seq analysis was performed on the infected wheat leaf tissues. Total RNA was extracted from three biological replicates at each of 5 time points during infection (12, 24, 48, 72, and 96 h post-inoculation (HPI)) for each isolate separately, as well as three replicates of a mock-inoculated control (0 HPI). RNA-seq libraries were constructed for each of the 33 RNA samples and sequenced using Illumina HiSeq 2500, generating between 36 and 50 million quality-trimmed single 100-bp reads for each RNA-seq library (Additional file 1: Table S1).

To assess the level of host gene expression, all reads were first aligned to the reference genome of wheat [32]. To assure that RNA-seq reads are mapped to the correct copies of genes within the polyploid genome, the parameters of the Tophat aligner [37] were optimized using a subset of reads mapping to a homoeologous set of 100 genes present in single copy in each of the wheat genomes. The alignment parameters were selected to maximize the proportion of reads mapping uniquely to each of the duplicated homoeologs. The final analyses were performed using the program settings that allowed for mapping RNA-seq reads in the training dataset with an error rate of less than 1% (Additional file 1: Figure S1).

RNA-seq reads that did not map to the wheat genes, and therefore enriched for fungal sequences, were combined to perform a *de novo* transcript assembly using Trinity v2.0.6 [33]. The combined *de novo* assembled transcripts together with the transcripts predicted in the SCCL genome [41] were used to annotate the MCCFC and RKQQC genomes using the PASA pipeline v2.0.1 [42] (Additional file 1: Table S3). In total 18,166 and 18,777 gene models were annotated within the MCCFC and RKQQC genomes, respectively. The predicted open reading frames from the annotated genes were used to extract 16,716 and 16,253 complete proteins from the MCCFC and RKQQC genomes, respectively (Additional file 1: Table S3).

**Fig. 1:**
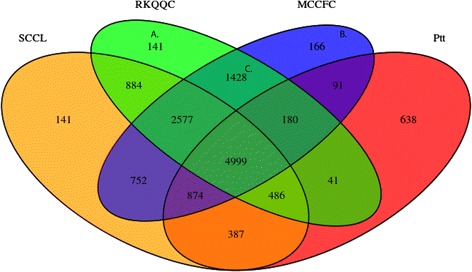
Venn diagram displays the results of OrthoMCL clustering. The analysis included 15,979, 16,716, and 16,253 proteins predicted in the SCCL, MCCFC, and RKQQC genomes, respectively. In addition, OrthoMCL clustering included 15,685 proteins predicted in the *Puccinia triticina* f.sp. *tritici* (Ptt) genome. Numbers represent the counts of orthologous/paralogous protein groups. Groups composed exclusively of proteins from the RKQQC (**a**) and MCCFC genome (**b**) included 315 and 361 proteins, respectively

The proteomes of both isolates were compared with the 15,979 publically available proteins predicted in the SCCL genome and the 15,685 proteins predicted in the *Puccinia triticina* (*Ptt*) genome [41, 43]. These proteomes were used in an all-against-all BLASTP comparison followed by protein clustering using OrthoMCL to identify the groups of orthologous and paralogous genes (Table 4) [34]. In total, 64,633 proteins from the four genomes were clustered into 13,785 groups containing varying number of orthologous and paralogous proteins (Fig. 1). Out of these groups, 1,086 were composed of proteins from only one genome, which with the 7,962 ungrouped proteins results in 11,190 proteins that were unique to one of the four sequenced fungal genomes (Table 4). These unique proteins included 1,726 and 1,703 proteins from the MCCFC and RKQQC genomes, respectively (Table 4). Additionally, 1,428 groups were found to contain proteins from only the MCCFC and RKQQC genomes, and respectively included 1,633 and 1,619 proteins (Fig. 1). In total, 3,359 and 3,322 novel proteins were identified in the MCCFC and RKQQC genomes, respectively. The sequences of these proteins were compared to the proteins predicted in the *Pgt* PANaus pan-genome assembly compiled from five Australian *Pgt* isolates  [16]. In total, 2,594 (78%) of the novel RKQQC proteins and 2,617 (77.9%) of the novel MCCFC proteins showed sequence similarity to the proteins in the PANaus dataset [16]. The identification of homologous sequences in these independently sequenced *Pgt* isolates suggests that the isolate-specific genes identified in the RKQQC and MCCFC genomes are unlikely to be the result of contamination from other DNA sources, but rather represent true presence-absence polymorphisms among the *Pgt* isolates. Additionally, 728 proteins in the RKQQC genome and 742 proteins in the MCCFC genome showed no significant similarity to any *Pgt* proteins and likely represent unique genes within these newly sequenced *Pgt* isolates. Out of these novel proteins, 626 from the RKQQC genome and 625 from the MCCFC genome showed similarity to the known PFAM domains (Additional file 3: Table S4).

**Table 4:** Summary of OrthoMCL protein clustering results

Source	Combined	SCCL	MCCFC	RKQQC	*Puccinia triticinia*
Number of proteins used for clustering	64,633	15,979	16,716	16,253	15,685
Number of ungrouped proteins (unique proteins)	7,962	2,158	1,365	1,388	3,051
Number of proteins clustered into ortholog/paralog groups	56,671	13,821	15,351	14,865	12,634
Number of ortholog/paralog groups	13,785	11,100	11,067	10,736	7,696
Number of unique (single genome) groups	1,086	141	166	141	638
Number of proteins in unique (single genome) groups	3,228	333	361	315	2,219
Maximum number of proteins/group	260	107	61	69	106
Total number of unique proteins (unique groups + ungrouped proteins)	11,190	2,491	1,726	1,703	5,270

An alternative approach to assessing the gene content divergence between the newly sequenced *Pgt* isolates was based on the combined comparative analysis of genomes and RNA-seq data. For this purpose, the RNA-seq data from both isolates were separately aligned against the SCCL reference transcripts. RNA-seq reads from the RKQQC and MCCFC infected leaf tissues were successfully aligned to 10,154 and 10,437 SCCL genome gene models, respectively, and individual transcript abundances were calculated for each isolate. A conservative approach was taken to qualify genes as present or absent in the sequenced genomes. A gene annotated in the SCCL genome was considered present in a newly sequenced isolate if it showed expression (FPKM > 0) within that isolate and/or if it resided within an orthologous group with a protein from that isolate. For a gene annotated in the SCCL genome to be considered absent from the genomes of RKQQC or MCCFC it would have to lack any detectable expression from that isolate, and have no detected orthologs from that isolate within the OrthoMCL dataset. In total, 810 genes (5.1%) were found to be present in the RKQQC genome and absent in the MCCFC genome. Conversely, 825 SCCL genes (5.2%) were present in the MCCFC genome and absent in the RKQQC genome. A total of 1,509 genes (9.4%) annotated in the SCCL genome were not detected in the transcript data from either isolate. The high degree of divergence in the gene contents of RKQQC, MCCFC, and SCCL genomes is consistent with the degree of divergence observed in other *Pgt* isolates as well as between isolates of other fungal pathogens [14, 16, 44]. Expectedly, the highest level of divergence in the gene content was found between the *Ptt* genome and the genomes of *Pgt* isolates.

### MCCFC and RKQQC Pgt races have distinct effector complements

**Fig. 2:**
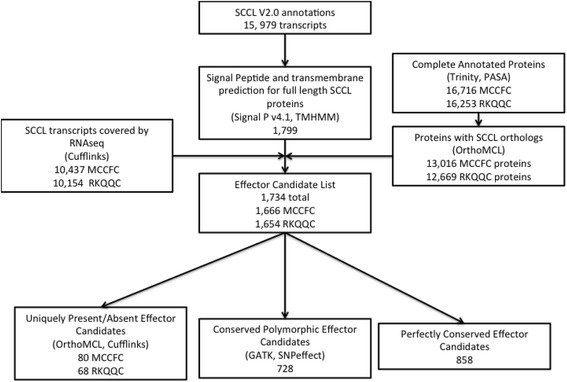
Diagram showing the pipeline for candidate effector classification into three groups. The SCCL gene models were compared with the genomic and transcriptomic sequence data generated for MCCFC and RKQQC. Effector candidates were categorized as unique, conserved polymorphic, or perfectly conserved between the two isolates

Biotrophic fungal pathogens interact with and manipulate their host plants through the use of secreted effector proteins. For this study, several criteria were used to identify and partition the likely effector candidates present in the genomes of the two *Pgt* isolates (Fig. 2). All 15,979 proteins predicted in the SCCL genome were screened for the presence of N-terminal signal peptide [45] and the absence of transmembrane domains outside the signal peptide region [35]. As described above, the OrthoMCL protein family information along with the RNA-seq data were used to identify those effector candidates that were present, and those that were absent from each isolate. Of the 1,799 secreted proteins identified in the SCCL genome, 1,586 were found present in both RKQQC and MCCFC, while 68 and 80 were found exclusively in either RKQQC or MCCFC, respectively, and 65 candidates were not detected in either isolate (Additional file 2: Table S2).

Using variant calling data, at least one non-synonymous mutation was found in 787 candidate effector-encoding genes. Based on these data the effector candidates were categorized into three groups (Additional file 2: Table S2). The first was composed of 148 candidates that were exclusively found in either the MCCFC or RKQQC genomes. Although it appears that these proteins are ostensibly dispensable for infection of the wheat cultivar Morocco, they are still high priority candidates because this type of presence/absence variation is the most likely to have an effect on the *Pgt* virulence. The second group of candidate effectors is composed of 728 genes that are conserved in both the MCCFC and RKQQC genomes yet show non-synonymous coding sequence variation between the isolates. These polymorphisms have the potential to alter the function of specific proteins and affect the interaction between the pathogen and host. The remaining 858 genes were conserved between the MCCFC, RKQQC, and SCCL genomes, containing only synonymous SNP variation in the coding sequences. These conserved genes likely represent a core group of effector candidates enriched for effectors that may be essential for successful fungal infection.

### Co-regulation of host and pathogen transcriptomes during the course of infection

To better understand the effects of effector complement divergence and conservation between the *Pgt* isolates on host gene co-regulation in the rust-wheat pathosystem, the time-resolved RNA-seq expression profiles of infected leaf tissues were generated. The RNA-seq reads mapped to the publically available wheat [32] and *Pgt* [41] reference genomes were used to obtain the FPKM values for their respective gene models at each of the six infection time points 0, 12, 24, 48, 72, and 96 HPI (NCBI GEO accession number GSE93015). The FPKM values were used to compare the joint expression of fungal (except 0 HPI) and wheat genes between different isolate treatments at the same time points, as well as across the time points of the same isolate treatment (Additional file 1: Figure S2). Based on the relative proportion of fungal reads mapped to the reference genome, both *Pgt* isolates demonstrated quite similar temporal patters of gene expression with the RKQQC race showing the slightly reduced proportion of mapped reads at 72 and 96 HPI (Additional file 1: Figure S3). However, the difference in the fraction of mapped reads at the 96 HPI time-point was not statistically significant.

In total 1,238 *Pgt* genes and 6,750 *T. aestivum* genes were identified as differentially expressed between the two isolate treatments and/or across the time course of infection for at least one isolate (FDR ≤ 0.05, log_2_-fold expression change ≥ 2) (Table 5). For the detailed analyses of expression profiles, the data was further filtered to retain only those genes that have data available for at least six biological replicates in the entire dataset, resulting in 1,054 *Pgt* genes and 3,877 wheat genes (Additional file 4: Tables S5 and S6). The pair-wise correlation analysis between the expression profiles of each fungal gene in the MCCFC and RKQQC datasets showed that the majority of genes (57%) have similar expression patterns during the course of infection with the Pearson correlation coefficient (PCC) above 0.5 (Additional file 1: Figure S4 and Additional file 5: Table S7). Comparison of the wheat expression profiles between the MCCFC and RKQQC datasets revealed that the substantial fraction of genes (71%) show similar patterns with the PCC > 0.5 (Additional file 1: Figure S4 and Additional file 5: Table S8).

**Table 5:** Wheat and *Pgt* genes differentially expressed between the *Pgt* isolates or across different time-points

Species	Differentially expressed (DE) between *Pgt* isolate treatments at specific time point					DE at all time points	DE across time series	Total DE genes non-redundant
	12 HPI	24 HPI	48 HPI	72 HPI	96 HPI			
*P. graminis*	317	308	257	533	571	153	707	1,238
*T. aestivum*	167	182	177	419	703	0	6562	6750

Further, we have functionally classified genes shared between the MCCFC and RKQQC datasets using the GO terms and assessed the average PCC for genes within each GO group (Additional file 5: Table S9 and S10). In *Pgt*, the most similar expression profiles were obtained for genes involved protein biosynthesis (GO:0043043, GO:0005840) and various metabolic processes (GO:1901135, GO:0019637), while the genes involved in the regulation of transcription (GO:1903506, GO:0003677) and RNA biosynthesis (GO:2001141) showed the lowest level of gene expression correlation. Among the wheat genes, those that were involved in protein biosynthesis (GO:0032544, GO:0008135, GO:0006412), stress response (GO:0009409, GO:0048583), transcription factor activity (GO:0001071) showed the high level of gene expression correlation between the MCCFC and RKQQC datasets. The lowest correlation values were found for genes involved in lipid transport (GO:0006869) and cellulose metabolism (GO:0030243).

**Fig. 3:**
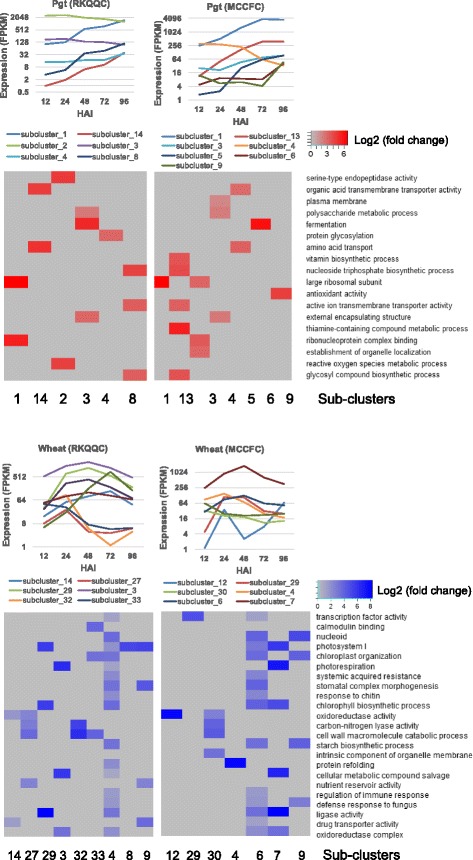
Clusters of co-expressed *Pgt* and wheat genes. Co-expressed gene clusters were obtained by k-means clustering of the expression data generated by the RNA-seq profiling of the MCCFC- and RKQQC-infected leaves. The GO term enrichment levels for each gene cluster are shown on the heat maps as the log2 fold changes compared to the genome-wide estimates

To obtain more detailed picture of the complex transcriptional events that occur in both plant and pathogen over the course of infection, we performed k-means gene clustering (Fig. 3) for each isolate-specific dataset (Additional file 6: Table S11). A number of gene clusters containing both the *Pgt* and wheat genes have been identified suggesting the connectedness of host’s and pathogen’s regulatory processes. The biological significance of each gene cluster was assessed by performing the GO term enrichment analysis (Additional file 6: Tables S12-S17). We have selected top 10 and 9 clusters for the wheat genes in the RKQQC and MCCFC datasets, respectively (Fig. 3). The host’s gene clusters in plants infected with different *Pgt* isolates were enriched for similar GO terms likely indicating the significance of associated pathways for plant-pathogen interaction. Among the identified clusters there were genes known for their involvement in response to the pathogen infection such as salicylic acid-dependent systemic acquired resistance [46], response to chitin [47], defense response to fungus, and the regulation of immune response (Fig. 3). The GO-enriched clusters also included genes involved in photorespiration and chloroplast organization that play critical role in the outcome of fungal infection [48]. Similar to the results of the transcriptome profiling of infected wheat tissues [24], the stress response pathways associated with salicylic acid- and jasmonic acid-induced metabolic processes were also over-represented in the wheat clusters.

The pathogen clusters generated for both the MCCFC and RKQQC datasets were enriched for genes with the oxidoreductase and antioxidant activities (Additional file 6: Tables S12-S17). Similar to previous study of the stripe rust infected wheat leaves [24], we also found over-representation of the genes involved in nucleic acid, protein and polysaccharide metabolism. The development of fungi was also associated with the increase in the transmembrane transport (Fig. 3) that was also demonstrated for *Fusarium oxysporum* during the colonization of *Medicago truncatula* host [49]. We have identified 7 clusters in the RKQQC dataset and 8 clusters in the MCCFC dataset enriched for the fungal genes encoding candidate effectors with the secretion signal peptides (Fig. 3, Additional file 6: Tables S14 and S17), consistent with the role of effectors in manipulating host’s responses to establish compatible interaction.

### Comparative analysis of GCNs identifies conserved and divergent sub-networks around specific gene ontologies

GCNs have been shown to be a powerful tool to characterize system level interactions between a host and a pathogen [50]. As with the K-means clustering, the *Pgt* isolate-specific GCNs were constructed using the FPKM expression values obtained for plant and pathogen genes that showed statistically significant changes over the experiment. To account for the non-scale free nature of the time-course RNA-seq data, a Graphical Gaussian model implemented in the R package GeneNet was used to calculate partial correlations between the expression profiles of each gene within the respective isolate treatment [38, 51]. Significant partial correlations (network edges) between genes (network nodes) were selected using the FDR cutoff value of 0.001 (Additional file 7: Tables S18 and S19).

The RKQQC and MCCFC race treatment networks contained 2,811 and 2,843 significantly connected nodes from both host and pathogen, linked respectively by 87,975 and 141,343 edges. The direct comparison of all significant edges between the isolate-specific networks revealed that 18,023 edges are conserved representing 20.5% of the RKQQC and 12.8% of the MCCFC treatment edges. Interestingly, the edge conservation was not equally distributed across the networks. The edges connecting certain groups of nodes show a much higher degree of network conservation than others. This variation in edge conservation is most apparent when dividing edges by the species-of-origin of nodes they connect. Between the two isolate-specific networks, edges linking wheat nodes to other wheat nodes were 13.8% conserved, edges connecting *Pgt* to *Pgt* nodes were 2.9% conserved, and those linking *Pgt* to wheat nodes were only 1.7% conserved (Table 6).

**Table 6:** The level of edge and node conservation between the isolate-specific GCNs

Types of network edges	Total edges	*Pgt*-wheat edges	*Pgt*-*Pgt* edges	Wheat-wheat edges	Total nodes	Wheat nodes	*Pgt* nodes
Conserved	18,023	957	1,131	15,935	1,471	1,062	409
	-	5.3%	6.3%	88.4%	-	72.2%	27.8%
Unique to RKQQC	69,952	17,215	19,491	33,246	2,788	1,875	913
	-	24.6%	27.9%	47.5%	-	67.3%	32.7%
Unique to MCCFC	123,320	38,763	17,939	66,618	2,842	2,031	811
	-	31.4%	14.6%	54.0%	-	71.5%	28.5%
Percent conservation	8.5%	1.7%	2.9%	13.8%	20.7%	21.4%	19.2%

**Fig. 4:**
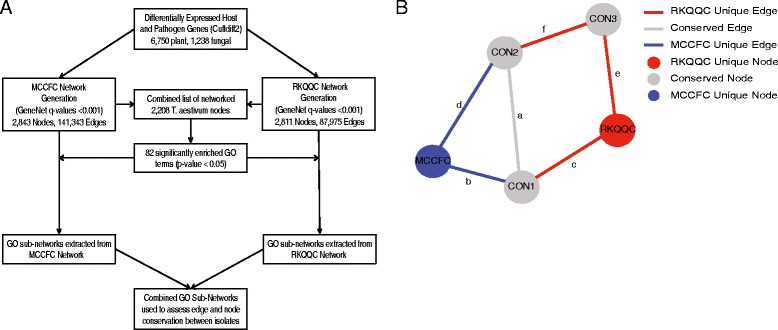
Comparison of *Pgt*-wheat gene co-expression networks. **a** A diagram of the analysis pipeline used to generate the combined GO sub-networks from each isolate-specific GCN. **b**. The example of combined network composed of three nodes the are conserved between both networks, and two nodes that are unique to the RKQQC- and MCCFC-specific networks. In this example, edges a, b, and c are first order edges for node CON1, while d, e, and f are second order edges for the same node. In this example, only edge a is conserved between the two isolate-specific networks. A sub-network for node CON1 contains nodes CON2, RKQQC, and MCCFC, but excludes CON3 because it doesn’t have a first order edge connecting it to CON1. The edges of the CON1 sub-network include edges a, b, c, and d, but exclude e and f because they connect with a node outside of the sub-network

To understand which biological processes are affected by each *Pgt* isolate in the susceptible host, all wheat genes within each isolate-specific GCN were functionally annotated using the Gene Ontology (GO) terms [52] (Fig. 4a). Significantly enriched GO terms were identified for three types of nodes in the GCNs: nodes connected by the RKQQC-specific edges, nodes connected by the MCCFC-specific edges, and the nodes connected by the conserved edges found in both GCNs. In total 82 GO terms were significantly enriched (Fisher exact test, *p*-value < 0.05) within one or more node groups (Additional file 1: Table S20). After removing redundancy inherent in the GO term hierarchy, this list was winnowed down to 51 groups of GO annotated wheat genes. To identify network modules that are associated with each enriched GO term, the conserved GO annotated genes were used to extract sub-networks from each isolate (henceforth, GO sub-networks). These sub-networks contained first order-connected nodes originating from wheat and *Pgt* including among other fungal genes the *Pgt* effector candidates. To identify the nodes and edges that were conserved or unique between the isolate-specific networks, combined sub-networks containing nodes from both isolate-specific GCNs were created by merging the GO sub-networks from each isolate based on the conserved nodes and edges (Fig. 4b).

**Fig. 5:**
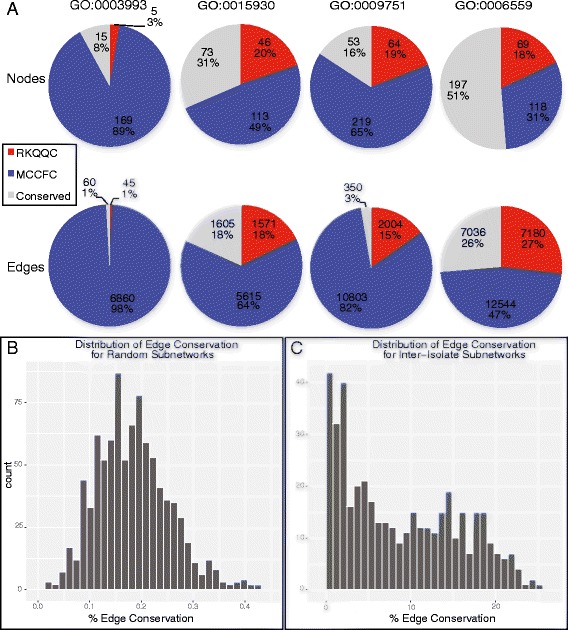
Wheat GO sub-networks vary in the degree of network conservation between the *Pgt* isolate treatments. **a** Examples of four different GO sub-networks, each including five wheat GO annotated genes, show a high degree of variability in both edge and node conservation between the two *Pgt* isolate treatments. **b** Each GO sub-network was randomly regenerated using the same number of nodes and edges observed in our experimentally reconstructed GCNs. For this purpose, sub-networks including five wheat nodes were randomly sampled 1,000 times, and produced a normal distribution of edge conservation values with a mean of 0.18%. **c** Five node sub-networks were randomly sampled from the real world data, and produced a multi-modal distribution of edge conservation values ranging from 0.05 to 24.81%

Comparisons of the combined sub-networks revealed that they vary widely in terms of the proportion of nodes and edges that are conserved (Fig. 5) (Additional file 1: Table S21). Some combined GO sub-networks showed a high degree of node and edge conservation, while others had mostly unique sets of nodes and edges (Fig. 5a). The proportion of conserved edges ranged from 0 to 26%, and the proportion of node conservation in the GO sub-networks ranged from 1.3 to 55%. Randomized networks, created by randomly regenerating all the edges of each isolate network yet preserving the same number of nodes and the total number of edges, showed significantly lower level of node/edge conservation pattern (Fig. 5b). The level of node and edge conservation was assessed for each of one thousand sub-networks that were created by randomly choosing sets of 5-nodes from both the randomized and the real-world isolate networks (Fig. 5b and c). The results of the permutations revealed that many of the GO sub-networks within the experimental dataset show a significantly higher degree of edge conservation than is expected between randomly generated networks of equal size and degree of connectedness. Furthermore, this edge conservation varied widely across the real-world sub-networks, with some sub-networks showing a high level of conservation and others showing little or no conservation (Fig. 5a). The observed heterogeneity in node/edge conservation across the real-world GCNs suggests that while certain host plant pathways can be modulated in a similar manner by both *Pgt* isolates, likely utilizing the virulence factors (effectors) shared by both *Pgt* isolates, other pathways might be affected by the virulence factors differentiating one *Pgt* isolate from another (Additional file 1: Table S21).

The *Pgt* effector candidates within the networks showed conservation in terms of the GO sub-networks they were associated with. Although the overall edge conservation between network conserved fungal effector candidates and wheat genes was relatively low (2.3%), the rate of network conserved effectors being associated with the wheat genes in the same GO term, on average, was 13.7%, and was as high as 32.7% for some candidates. Indeed, of the 126 *Pgt* effector candidates present in both isolate-specific networks, 100 (79.4%) were associated with the same GO sub-network in each isolate.

**Fig. 6:**
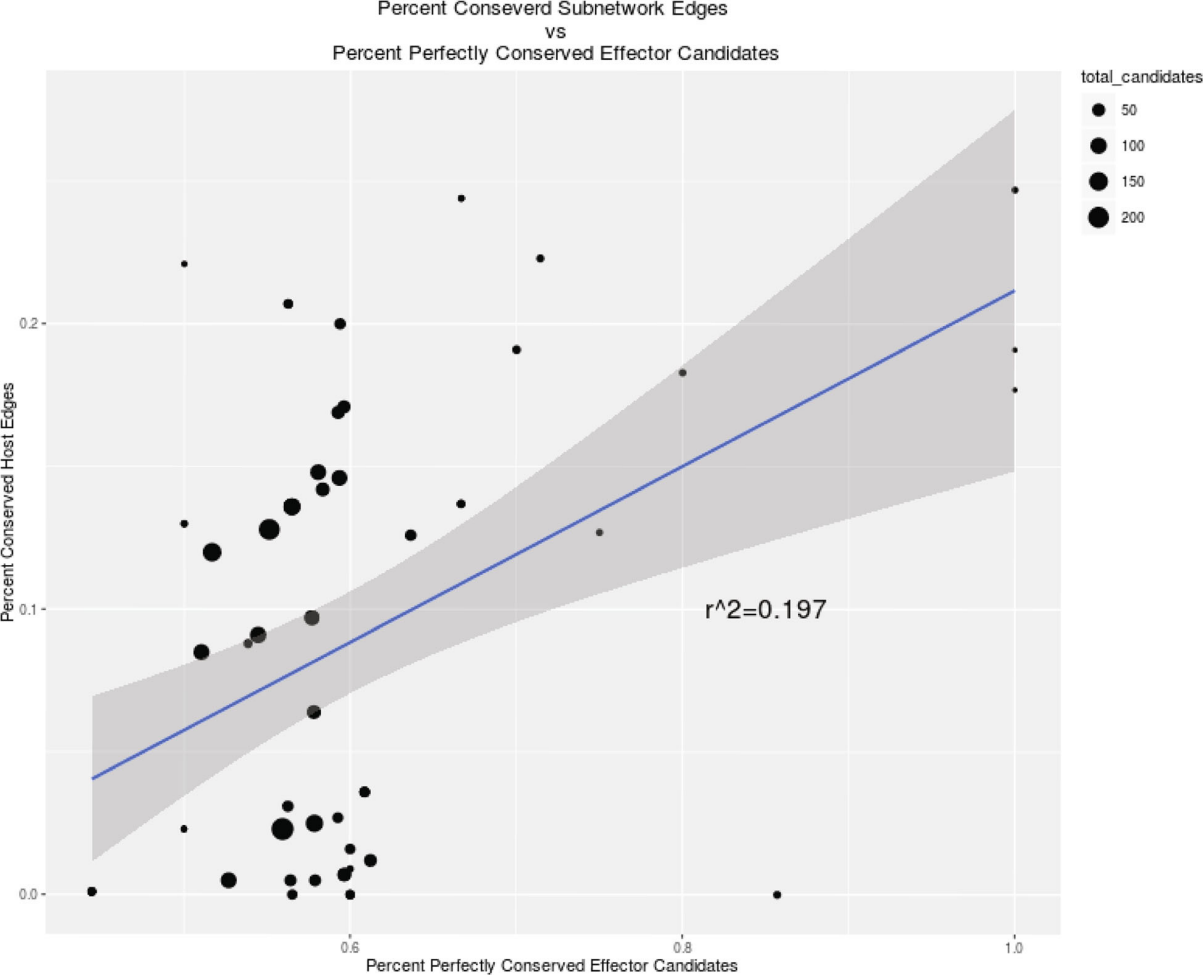
The relationship between GO sub-network edge conservation and proportion of perfectly conserved effectors. The percent edge conservation for each GO sub-network plotted against the percentage of perfectly conserved at the sequence level (as determined by genomic and transcriptomic sequence comparisons) effector candidates associated with that sub-network. Geometric point size reflects the total number of effector candidates associated with each sub-network. The best-fit regression line (*blue*) and standard error intervals (*dark grey*) are shown. Those sub-networks with a higher percentage of conserved effectors, particularly those with few effector candidates overall tend to have a higher degree of sub-network conservation

To investigate whether the degree of GO sub-network conservation is related to the sequence level conservation of effector candidates between the isolates, we compared the proportion conserved edges within each sub-network with the degree of DNA sequence conservation (as described in Fig. 2) for all effector candidates associated with each sub-network. We found that there is a significant positive correlation between the proportion of sequence conserved effector candidates associated with a specific GO sub-network and the level of edge conservation in that sub-network (Fig. 6) (R^2^ = 0.2). These results suggest that GO sub-networks with the higher levels of edge and node conservation between networks also show tendency to be associated with a higher proportion of effectors showing the high levels of sequence conservation. This trend has lead us to hypothesize that these effector candidates may be directly involved in modulating these biological pathways within the host plant, and that the degree of sequence divergence in the effector complements likely influences the regulation of host pathways.

### RKQQC and MCCFC isolate treatments produce distinct co-expression networks around salicylic acid response genes

The observed variation in the proportion of conserved host-pathogen edges among different GO sub-networks suggest the existence of convergent and divergent modes of evolution among the host-pathogen regulatory modules, where some modules are regulated by a conserved set of effectors and some are regulated by a divergent set of effectors. The latter examples include quite intriguing cases of the GO sub-networks, which are affected by distinct sets of effectors from different *Pgt* isolates.

**Fig. 7:**
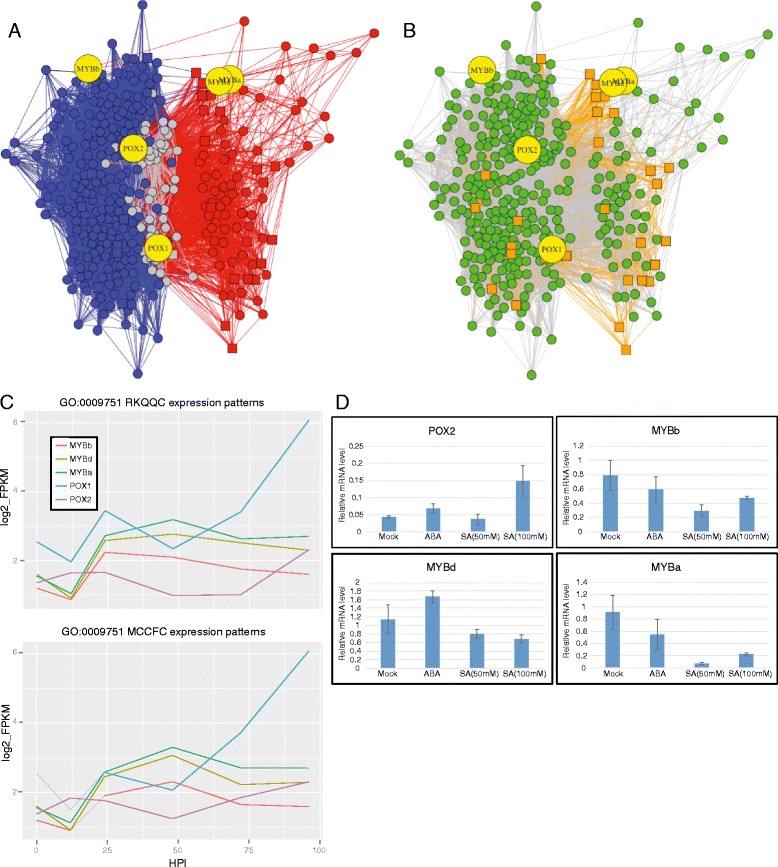
A comparison of the GO:0009751 co-expression sub-networks from each isolate. **a** The conserved (*grey*) nodes and edges, versus the nodes and edges that are unique to the RKQQC treatment (*red*) and MCCFC treatment (*blue*) are shown. The GO annotated genes themselves are highlighted as *yellow* nodes. **b** The candidate effector nodes within both sub-networks (*orange squares*), and their connections (*orange edges*) with the host plant nodes (*green*) are shown. **c** The log2 FPKM expression values of the 5 genes from GO:0009751, which do not show significant difference in expression under each isolate treatment. **d** Relative transcript abundance *T. aestivum* genes annotated as (GO:009751) responsive to salicylic acid. Transcript specific primers were used to perform qRT-PCR on mRNA extracted from wheat lines 18 h after salicylic acid (SA), abscisic acid (ABA), or mock treatments

Here, we have performed a more detailed analysis of one set of genes that produce a distinct sub-network structure within each isolate and include the salicylic acid (SA) responsive genes (GO:0009751) (Fig. 7a). Despite the fact that all of the five SA-responsive genes themselves showed similar expression profiles between the isolate treatments (Fig. 7c), relatively few of the other nodes and edges, including the effector-encoding genes, within the SA sub-network were conserved between the isolates (15.8% node conservation and 2.7% edge conservation (Fig. 7a, Additional file 1: Table S21). In other words, it seems that very different systematic changes caused by each isolate treatment can produce very similar results with regard to these SA-responsive genes. The low level of edge/node conservation suggests that the SA response pathway is modulated differently by each *Pgt* isolate, perhaps by deploying different sets of effectors (Fig. 7b). A total of 27 *Pgt* effector candidates were associated with the SA co-expression sub-network, with only 2 candidates (PGTG_18238 and PGTG_02185) consistently associated with the sub-network in both *Pgt* isolates.

The five GO:0009751 annotated genes are known for their association with the plant-fungal interaction. Three of the genes are homologous to the MYB transcription factors (Traes_5AS_7D519210E.5, Traes_5BS_D1C03C165.1, Traes_5DS_1EF547639.5) and thus have been designated MYBa, MYBb, and MYBd, respectively. All three of these genes showed protein sequence similarity with MYB59 (AT5G59780) and MYB48 (AT3G46130) from *Arabidopsis*. In *Arabidopsis*, these two transcription factors were shown to be specifically up regulated in response to SA treatment [53]. Additionally, the expression of AT5G59780 has also been shown to be responsive to chitin treatment [54] indicating that this gene is likely involved in the recognition of or response to pathogenic fungi.

The presence of these MYB transcription factors in the SA-responsive sub-network raised the possibility that they are involved in the co-regulation of gene expression within this sub-network. To investigate this possibility further we used Nsite program [55] to predict putative transcription factor binding sites within the promoter regions 3 Kb upstream of each wheat gene from the SA-responsive sub-network. A total of 50 MYB-responsive elements have been identified in the promoter regions of genes within the sub-network, a significant enrichment relative to the genes in the GCNs as a whole (Fisher’s exact test, P-value = 0.017) (Additional file 8: Table S22).

The two other GO:0009751 annotated genes Traes_2AL_6A8D574C4.1 and Traes_2AL_52C4B6996.2 showed amino acid sequence similarity to caleosins and peroxygenases from other plant species and were designate POX1 and POX2, respectively (Fig. 7a). Peroxygenases are part of the oxylipin metabolic pathway, which is known to generate anti-fungal compounds [56, 57]. RD20, a caleosin/peroxygenase from *Arabidopsis*, has been shown to respond to salicylic acid and increase plant resistance to pathogenic fungi via the oxylipin pathway [58, 59]. Furthermore, oxylipin metabolism has also been shown to play an important role in the fungal pathogenesis of wheat. Lipoxygenases, another group of enzymes involved in the oxylipin pathway, were recently shown to function in the wheat defense response against *Fusarium graminearum* [60]. Therefore, the finding of 7 transcripts (Ta2alMIPSv2Loc183543.1, Traes_2BL_77148B8D8.1, Traes_2DL_CE85DC5C0.1, Traes_2DL_B5B62EE11.2, Traes_5BS_060785740.2, Traes_5DS_E8892706A.2, and Traes_6DS_E66547E66.2) with strong similarity to lipoxygenases from wheat and other grasses in the combined SA-responsive sub-network provides further support in the validity of the constructed GCN, and suggests that the oxylipin metabolic pathway possibly plays important roles in the interaction between wheat and *Pgt.*

To confirm responsiveness, either positive or negative, of these genes to SA in wheat, their expression levels were assessed in the wheat seedlings of cultivar Morocco treated with the solutions of SA. Using qRT-PCR we compared the expression for four of the GO:0009751 annotated genes after SA treatment against mock and abscisic acid-treated controls (Fig. 7d). All four genes showed a significant change in expression, either positive or negative, in response to SA treatment (*P*-value < 0.05).

These data are a further indication that the homology-based GO annotations for these genes likely reflect true functional roles in the wheat SA response pathway. The SA mediated repression of the three wheat MYB transcription factors is consistent with the transcriptional changes observed in a minority of Arabidopsis MYBs after treatment with SA [53]. Conversely, the consistent and slightly increased expression of these MYBs over the course of both compatible interactions seems to support their potential for having roles in the plant defense response against *Pgt*.

## Discussion

Here, we have performed the comparative analyses of genomic and transcriptomic data for two North American *Pgt* isolates showing distinct virulence profiles on the panel of wheat lines carrying known stem rust resistance genes [40]. The differences in the virulence profiles of these isolates were reflected by substantial inter-isolate gene content variation consistent with that observed in other plant pathogenic fungi [14, 16, 61, 62]. Due to their direct interaction with the host factors, the patterns of inter-isolate sequence and presence/absence variation in the effector encoding genes have been one of the major focuses of recent plant-pathogen interaction studies [5, 9, 10]. Consistent with the previous genomic comparisons of pathogenic fungi [10, 14, 63], in our study, the substantial fraction of candidate secreted proteins showed accumulation of non-synonymous mutations (42%) and PAV (4.6%). The “arms-race” model suggests that the preferential retention of genomic mutations promoting effective virulence on a diverse set of the host resistance genes results in the diversification of effector encoding genes [64, 65]. However, while the evidence for fast diversifying selection has been reported for effectors [10, 66], it remained unclear how divergent complement of effectors in different isolates maintain the ability to establish compatible interaction on the same hosts. The comparative analyses of the gene co-expression networks and *Pgt* genomes reported here provided some new insights into the possible effects of isolate divergence on the establishment of compatible host-pathogen interactions.

We found the high level of heterogeneity in the degree of inter-isolate node and edge conservation across different GO sub-networks when both host and pathogen genes are considered. However, the enrichment of the same wheat GO terms within both isolate-specific networks indicates that, in spite of genomic divergence, these two isolates tend to utilize the same host pathways for establishing a compatible interaction. These conserved pathways likely play critical roles in compatible interaction and include GO terms known to be associated with plant-pathogen interaction, including lyase activity (GO:0016829) [67], genes associated with DNA catabolism (GO:0016798) [68, 69], and salicylic acid response genes (GO:009571). The levels of host GO sub-network edge conservation showed a positive correlation (R^2^ = 0.2) with the proportion of effector candidates showing high levels of sequence conservation between the *Pgt* isolates suggesting that the host pathways targeted by the same effectors show tendency to be regulated in a similar manner. Though sequence conservation explains only 20% of network edge conservation, it is worth noting that these associations are likely confounded both by the presence of non-functionally relevant variations between the two effector compliments, as well as the presence of multiple redundant effectors targeting the same pathway in both isolates. For some sub-networks, divergence in the effector complement appeared to have relatively minor effects on host sub-network structure and conservation. Indeed, the presence of host sub-networks that show a high level of node conservation but do not share common fungal nodes suggest the functional convergence of divergent sets of fungal genes to the same pathways. Taken together, these results suggest that the host networks utilized by the stem rust fungal pathogen to establish compatible interaction appear to be relatively robust to changes in the pathogen’s effector complement. This funding is reminiscent of the observation made for two *Arabidopsis* pathogens spanning the eukaryote-eubacteria divergence [70]. In that study, the divergent sets of the independently evolved effectors from bacterial and fungal pathogens showed the evidence of convergence onto a limited number of cellular targets. Our data indicate that even at the intra-species level pathogen populations likely maintain divergent sets of effectors capable of targeting the same host pathways. This functional redundancy may play an important role in the dynamic of the “arms-race” between host and pathogen by creating conditions where mutations in a single effector will not have a major effect on its ability to infect the host.

The sub-network annotated as “response to salicylic acid” (GO:009571) is one of the pathways that displayed a low degree of host sub-network conservation between the two isolate treatments, and included additional genes with characterized roles in SA-mediate plant defense pathways. Despite the fact that all five GO:009571 annotated genes were present in both networks and not themselves differentially expressed between isolate treatments, the other co-expressed plant and fungal genes within with the sub-networks were different. The validity of the connections within these sub-networks is affirmed by a significant enrichment in the number of MYB transcription factor binding elements in the promoter regions of the SA sub-network nodes. Further affirmation comes from the presence of seven lipoxygenase homologs within the SA sub-networks. These genes function downstream of peroxygenase in the oxylipin pathway and have been shown to play important roles in plant-fungal interactions [57, 59, 60, 71, 72]. Peroxygenases themselves are known to play roles in plant defense, and as such both POX1 and POX2 within the sub-networks were also annotated with the GO terms “response to fungus” (GO:0009620) and “defense response to fungus” (GO:0042831). In light of what is known about the genes in the GO sub-networks it is perhaps unsurprising that these same five SA-responsive genes are modulated in a similar manner by two different *Pgt* isolates*.*

## Conclusions

Understanding how specific *Pgt* effectors are able to cause these types of changes within a host plant is an important factor when designing resistant crop varieties and monitoring the evolution of virulent pathogen populations [62, 73]. Though it is difficult to determine exactly which fungal genes are responsible for mediating specific transcriptional changes seen in the host during infection, the comparative network analysis in this study has identified effector candidates associated with specific molecular pathways within the host. The presented results suggest that the diversification of the complement of secreted effectors in different *Pgt* isolates can serve as the basis for diverse strategies utilized by a pathogen to establish compatible interaction with its host. While different isolates tend to exploit limited number of host biological pathways for infection, the mode of interaction implemented by diverged isolates can vary for different pathways. Some pathways appear to be manipulated by a conserved set of effectors while others involve effectors that are either isolate-specific or diverged. The functional convergence of secreted effectors onto some of the same pathways likely is a strong indicator of the importance of these pathways in compatible interaction. These data also indicate that by further expanding the number of characterized genetically diverse *Pgt* isolates and wheat lines, it should be possible to create a detailed functional map of host’s biological pathways and associated repertoire of effectors that promote compatible interaction between *Pgt* and wheat. The knowledge of these pathways, especially those that are associated with the conserved set of effectors showing limited variation across multiple fungal isolates, and, are therefore most likely critical for compatible interaction, will help to guide crop improvement efforts through the utilization of modern biotechnological and genomics-assisted breeding strategies.

## Additional files

Additional file 1: File contains 2 supplementary figures and 4 supplementary tables. **Figure S1.** The effects of different TopHap alignment parameters on the proportion of RNA-seq reads misaligned to single copy homoeologous genes in the wheat genome. **Figure S2.** Distribution of log10 FPKM values for all genes in each biological replicate. **Figure S3.** Proportion of RNA-seq reads for RKQQC (orange) and MCCFC (blue) datasets mapped to the public SCCL reference genome. Standard error for each time-point is shown. **Figure S4.** Distribution of Pearson correlation coefficient (PCC) values estimated for the same gene pairs by comparing the expression values from the MCCFC dataset with those in the RKQQC dataset. The PCC value for wheat and *Pgt* genes are shown in grey and red, respectively. **Table S1.** Summary of next-generation sequence data generated for this study. Data is available from the NCBI SRA BioProject PRJNA347320. **Table S3.** Summary of genomic assemblies, genome annotations using the PASA pipeline and BUSCO assembly quality assessments for each *P. graminis* isolate. **Table S20.** Summary of BLAST2GO enrichment analysis of over represented wheat gene ontology terms in the three network edge conservation groups: MCCFC-specific, RKQQC-specific and conserved. **Table S21.** Summary of metadata for the combined GO sub-networks. (DOCX 960 kb)

Additional file 2: File contains table with expression values and genomic conservation status for effectors. **Table S2.** Combined expression and genomic conservation data for all effector candidates from the SCCL gene models. (XLSX 3942 kb)

Additional file 3: File contains the list of novel *Pgt* genes and their PFAM annotation. **Table S4.** PFAM domains identified in the novel genes discovered in the MCCFC and RKQQC genomes. (XLSX 144 kb)

Additional file 4: File contains two tables with the gene expression values for both MCCFC and RKQQC datasets that were used for K-mean clustering. **Table S5.** Expression values of both *Pgt* and wheat genes in the RKQQC dataset. **Table S6.** Expression values of both *Pgt* and wheat genes in the MCCFC dataset. (XLSX 1757 kb)

Additional file 5: The file contains the estimates of Pearson correlation coefficients obtained for each gene or groups of genes from the same GO terms using the inter-isloate comparison of expression profiles. **Table S7.** Pearson correlation coefficients estimated for the same Pgt genes by comparing the expression values from the MCCFC datasets with those in the RKQQC dataset. **Table S8.** Pearson correlation coefficients estimated for the same wheat genes by comparing the expression values from the MCCFC datasets with those in the RKQQC dataset. **Table S9.** Pearson correlation coefficients estimated for the wheat GO terms by comparing expression values from the MCCFC datasets with those in the RKQQC dataset. **Table S10.** Pearson correlation coefficients estimated for the Pgt GO terms by comparing expression values from the MCCFC datasets with those in the RKQQC dataset. (XLSX 169 kb)

Additional file 6: File contain the results of k-means clustering and GO term enrichment analyses. **Table S11.** K-mean clustering of *Pgt* and wheat genes. **Table S12.** GO term enrichment for clusters generated using wheat genes expressed in the leaves inoculated with the *Pgt* RKQQC race. **Table S13.** GO term enrichment for clusters generated using Pgt genes expressed in the leaves inoculated with the *Pgt* RKQQC race. **Table S14.** Enrichment of effector encoding genes in gene clusters generated using the RKQQC dataset. **Table S15.** GO term enrichment for clusters generated using wheat genes expressed in the leaves inoculated with the *Pgt* MCCFC race. **Table S16.** GO term enrichment for clusters generated using Pgt genes expressed in the leaves inoculated with the *Pgt* MCCFC race. **Table S17.** Enrichment of effector encoding genes in gene clusters generated using the MCCFC dataset. (XLSX 359 kb)

Additional file 7: The file contains edges of GCNs developed or RKQQC and MCCFC datasets. **Table S18.** Edges of RKQQC-specific GCN. **Table S19.** Edges of MCCFC-specific GCN. (XLSX 914 kb)

Additional file 8: The file contains the list of MYC transcription factor binding elements from the SA-responsive sub-network. **Table S22.** MYB-responsive elements in the promoter regions of the SA-associated subnetwork (GO: 0009751) (XLSX 43 kb)

## Abbreviations

ABA: Abscisic acid
cv.: Cultivar
DE: Differentially expressed
FDR: False discovery rate
FPKM: Fragments per Kilobase of transcript per Million fragments mapped
GCN: Gene Co-expression networks
GO: Gene ontology
HPI: Hours post-inoculation
IGF: Integrated genomics facility
PCC: Pearson correlation coefficient
PE: Paired-end
Pgt: Puccinia graminis f. sp. tritici
Ptt: Puccinia triticina
SA: Salicylic acid
