# Supplementary material for: Divergent and convergent modes of interaction between wheat and Puccinia graminis f. sp. tritici isolates revealed by the comparative gene co-expression network and genome analyses
Source: BMC Genomics. 2017 Apr 12;18:291. doi: 10.1186/s12864-017-3678-6 (PMC5389088; doi:10.1186/s12864-017-3678-6)
Supplement: Supplementary file 1 — File contains 2 supplementary figures and 4 supplementary tables. Figure S1. The effects of different TopHap alignment parameters on the proportion of RNA-seq reads misaligned to single copy homoeologous genes in the wheat genome. Figure S2. Distribution of log10 FPKM values for all genes in each biological replicate. Figure S3. Proportion of RNA-seq reads for RKQQC (orange) and MCCFC (blue) datasets mapped to the public SCCL reference genome. Standard error for each time-point is shown. Figure S4. Distribution of Pearson correlation coefficient (PCC) values estimated for the same gene pairs by comparing the expression values from the MCCFC dataset with those in the RKQQC dataset. The PCC value for wheat and Pgt genes are shown in grey and red, respectively. Table S1. Summary of next-generation sequence data generated for this study. Data is available from the NCBI SRA BioProject PRJNA347320. Table S3. Summary of genomic assemblies, genome annotations using the PASA pipeline and BUSCO assembly quality assessments for each P. graminis isolate. Table S20. Summary of BLAST2GO enrichment analysis of over represented wheat gene ontology terms in the three network edge conservation groups: MCCFC-specific, RKQQC-specific and conserved. Table S21. Summary of metadata for the combined GO sub-networks. (DOCX 960 kb) [file 12864_2017_3678_MOESM1_ESM.docx]

Supplementary Material

**Divergent and convergent modes of wheat-pathogen interaction revealed by the comparative gene co-expression network and genome analyses of *Puccinia graminis f. sp. tritici* isolates.**

William B. Rutter, Andres Salcedo, Alina Akhunova, Shichen Wang, Hanquan Liang, Robert L. Bowden, and Eduard Akhunov*

*** Correspondence:** Corresponding Author: eakhunov@ksu.edu

# Supplementary Figures and Tables

## Supplementary Figures

**Figure S1.** The effects of different TopHap alignment parameters on the proportion of RNA-seq reads misaligned to single copy homoeologous genes in the wheat genome.


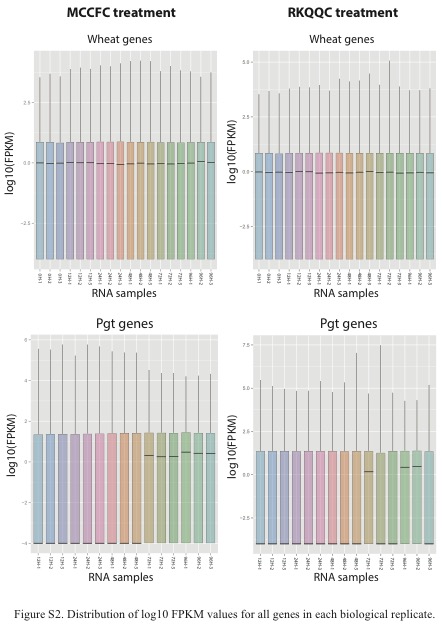


**Figure S2.** Distribution of log10 FPKM values for all genes in each biological replicate.

**Figure S3.** Proportion of RNA-seq reads for RKQQC (orange) and MCCFC (blue) datasets mapped to the public SCCL reference genome. Standard error for each time-point is shown.


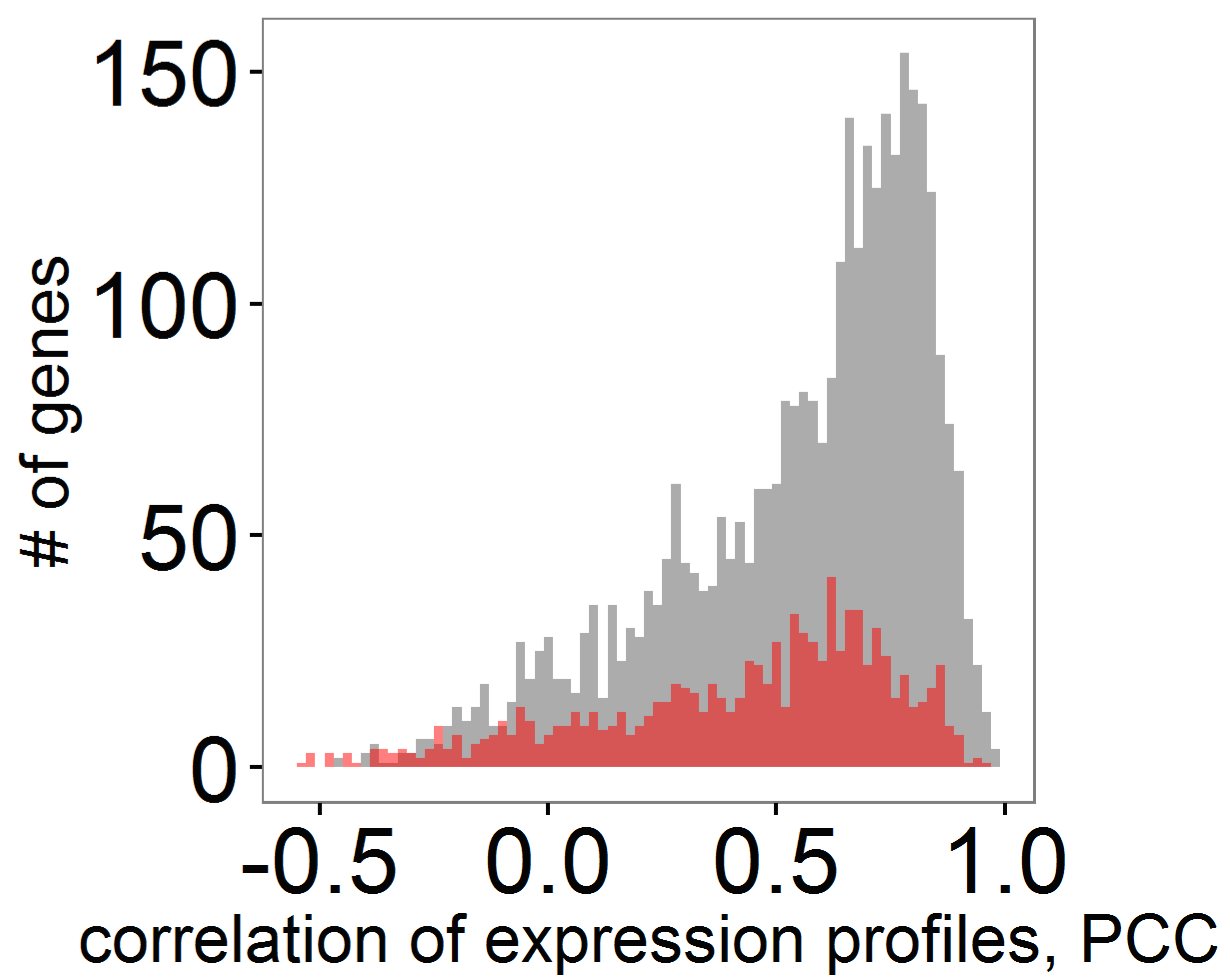


**Figure S4.** Distribution of Pearson correlation coefficient (PCC) values estimated for the same gene pairs by comparing the expression values from the MCCFC dataset with those in the RKQQC dataset. The PCC value for wheat and *Pgt* genes are shown in grey and red, respectively.

## Supplementary Tables

**Table S1.** Summary of next-generation sequence data generated for this study. Data is available from the NCBI SRA BioProject PRJNA347320.

| **Sample^a^** | **Data type** | **Technology** | **Read type** | **Num. reads** | **NCBI SRA ID** |
| --- | --- | --- | --- | --- | --- |
| Morocco_0h-rep1 | RNA-Seq | Illumina | 1x100 bp | 47,860,040 | SAMN05859607 |
| Morocco_0h-rep2 | RNA-Seq | Illumina | 1x100 bp | 44,199,292 | SAMN05859608 |
| Morocco_0h-rep3 | RNA-Seq | Illumina | 1x100 bp | 46,951,661 | SAMN05859609 |
| Morocco-RKQQC_12h-rep1 | RNA-Seq | Illumina | 1x100 bp | 52,687,795 | SAMN05859610 |
| Morocco-RKQQC_12h-rep2 | RNA-Seq | Illumina | 1x100 bp | 52,443,831 | SAMN05859611 |
| Morocco-RKQQC_12h-rep3 | RNA-Seq | Illumina | 1x100 bp | 53,534,290 | SAMN05859612 |
| Morocco-RKQQC_24h-rep1 | RNA-Seq | Illumina | 1x100 bp | 50,325,713 | SAMN05859613 |
| Morocco-RKQQC_24h-rep2 | RNA-Seq | Illumina | 1x100 bp | 50,345,705 | SAMN05859614 |
| Morocco-RKQQC_24h-rep3 | RNA-Seq | Illumina | 1x100 bp | 51,479,129 | SAMN05859615 |
| Morocco-RKQQC_48h-rep1 | RNA-Seq | Illumina | 1x100 bp | 52,875,178 | SAMN05859616 |
| Morocco-RKQQC_48h-rep2 | RNA-Seq | Illumina | 1x100 bp | 46,732,491 | SAMN05859617 |
| Morocco-RKQQC_48h-rep3 | RNA-Seq | Illumina | 1x100 bp | 48,784,559 | SAMN05859618 |
| Morocco-RKQQC_72h-rep1 | RNA-Seq | Illumina | 1x100 bp | 42,120,687 | SAMN05859619 |
| Morocco-RKQQC_72h-rep2 | RNA-Seq | Illumina | 1x100 bp | 42,467,874 | SAMN05859620 |
| Morocco-RKQQC_72h-rep3 | RNA-Seq | Illumina | 1x100 bp | 36,590,426 | SAMN05859621 |
| Morocco-RKQQC_96h-rep1 | RNA-Seq | Illumina | 1x100 bp | 36,124,098 | SAMN05859622 |
| Morocco-RKQQC_96h-rep2 | RNA-Seq | Illumina | 1x100 bp | 37,957,813 | SAMN05859623 |
| Morocco-RKQQC_96h-rep3 | RNA-Seq | Illumina | 1x100 bp | 44,387,711 | SAMN05859624 |
| Morocco-MCCFC_12h-rep1 | RNA-Seq | Illumina | 1x100 bp | 45,470,332 | SAMN05859625 |
| Morocco-MCCFC_12h-rep2 | RNA-Seq | Illumina | 1x100 bp | 43,263,006 | SAMN05859626 |
| Morocco-MCCFC_12h-rep3 | RNA-Seq | Illumina | 1x100 bp | 44,612,673 | SAMN05859627 |
| Morocco-MCCFC_24h-rep1 | RNA-Seq | Illumina | 1x100 bp | 45,091,502 | SAMN05859628 |
| Morocco-MCCFC_24h-rep2 | RNA-Seq | Illumina | 1x100 bp | 47,333,740 | SAMN05859629 |
| Morocco-MCCFC_24h-rep3 | RNA-Seq | Illumina | 1x100 bp | 44,657,311 | SAMN05859630 |
| Morocco-MCCFC_48h-rep1 | RNA-Seq | Illumina | 1x100 bp | 52,226,755 | SAMN05859631 |
| Morocco-MCCFC_48h-rep2 | RNA-Seq | Illumina | 1x100 bp | 49,590,601 | SAMN05859632 |
| Morocco-MCCFC_48h-rep3 | RNA-Seq | Illumina | 1x100 bp | 49,584,374 | SAMN05859633 |
| Morocco-MCCFC_72h-rep1 | RNA-Seq | Illumina | 1x100 bp | 48,177,593 | SAMN05859634 |
| Morocco-MCCFC_72h-rep2 | RNA-Seq | Illumina | 1x100 bp | 50,100,720 | SAMN05859635 |
| Morocco-MCCFC_72h-rep3 | RNA-Seq | Illumina | 1x100 bp | 49,345,816 | SAMN05859636 |
| Morocco-MCCFC_96h-rep1 | RNA-Seq | Illumina | 1x100 bp | 54,067,813 | SAMN05859637 |
| Morocco-MCCFC_96h-rep2 | RNA-Seq | Illumina | 1x100 bp | 46,629,166 | SAMN05859638 |
| Morocco-MCCFC_96h-rep3 | RNA-Seq | Illumina | 1x100 bp | 37,595,167 | SAMN05859639 |
| RKQQC | DNA-Seq | Illumina-Miseq | 2x300 bp | 31,390,809 | SAMN05859640 |
| MCCFC | DNA-Seq | Illumina-Miseq | 2x300 bp | 34,145,259 | SAMN05859641 |
| RKQQC | DNA-Seq | Pacific Bioscience | | 264,876 | SAMN05859642 |
| MCCFC | DNA-Seq | Pacific Bioscience | | 145,823 | SAMN05859643 |

a - the sample naming scheme as follows: Wheat line – *Pgt* race – Time in hours after infection – Biological replicate.

**Table S2 (see Additional File 2):** Table containing combined expression and genomic conservation data for all effector candidates from the SCCL gene models.

**Table S3:** Summary of genomic assemblies, genome annotations using the PASA pipeline and BUSCO assembly quality assessments for each *P. graminis* isolate.

| **Pgt isolates** | **MCCFC** | **RKQQC** | **SCCL_V2 (super contigs)** |
| --- | --- | --- | --- |
| Contigs | 28091 | 28502 | 4557 |
| Min length | 300 | 300 | 542 |
| Max length | 99632 | 80503 | 3081398 |
| Total length | 93295578 | 107317715 | 88644628 |
| N50 | 7133 | 6292 | 964966 |
| N75 | 3313 | 3357 | 428160 |
| Contigs (>= 1000 bp) | 16990 | 22341 | 392 |
| Contigs (>= 5000 bp) | 5491 | 7040 | 378 |
| Contigs (>= 10000 bp) | 2044 | 2087 | 265 |
| Contigs (>= 25000 bp) | 238 | 76 | 185 |
| Contigs (>= 50000 bp) | 20 | 2 | 170 |
| Total length (>= 1000 bp) | 86554594 | 103327860 | 88644628 |
| Total length (>= 5000 bp) | 58597646 | 64644919 | 88580633 |
| Total length (>= 10000 bp) | 34546836 | 30134859 | 87785950 |
| Total length (>= 25000 bp) | 8240388 | 2398169 | 86593993 |
| Total length (>= 50000 bp) | 1259490 | 131991 | 86098137 |
| **Annotations (PASA pipeline)** | | | |
| Total valid transcript alignments | 63406 | 58038 | NA |
| Number of assemblies | 22046 | 21926 | NA |
| Number of subclusters (genes) | 18166 | 18777 | NA |
| Total number of predicted peptides | 16716 | 16253 | 15979 |
| Average length of predicted peptides | 396.56 | 367.83 | 1187.4 |
| **BUSCO analysis** | | | |
| Complete Single-copy BUSCOs | 1060(73%) | 907(63%) | 1231(85%) |
| Complete Duplicated BUSCOs | 326(22%) | 334(23%) | 242 (16%) |
| Fragmented BUSCOs | 142(9.8%) | 201(13%) | 116 (8%) |
| Missing BUSCOs | 236(16%) | 330(22%) | 91(6.3%) |
| Number of genes searched for | 1438 | 1438 | 1438 |

**Table S4 (see Additional File 3):** PFAM domains identified in the novel genes discovered in the MCCFC and RKQQC genomes.

**Table S5 (see Additional File 4).** Expression values of both Pgt and wheat genes in the RKQQC dataset.

**Table S6 (see Additional File 4).** Expression values of both Pgt and wheat genes in the MCCFC dataset.

**Table S7 (see Additional File 5).** Pearson correlation coefficient estimated for the same Pgt genes by comparing the expression values from the MCCFC datasets with those in the RKQQC dataset.

**Table S8 (see Additional File 5).** Pearson correlation coefficient estimated for the same wheat genes by comparing the by comparing the expression values from the MCCFC datasets with those in the RKQQC dataset.

**Table S9 (see Additional File 5).** Pearson correlation coefficient estimated for the wheat GO terms by comparing the by comparing the expression values from the MCCFC datasets with those in the RKQQC dataset.

**Table S10 (see Additional File 5).** Pearson correlation coefficient estimated for the Pgt GO terms by comparing the by comparing the expression values from the MCCFC datasets with those in the RKQQC dataset.

**Table S11 (see Additional File 6).** K-mean clustering of *Pgt* and wheat genes.

**Table S12 (see Additional File 6).** GO term enrichment for clusters generated using wheat genes expressed in the leaves inoculated with the *Pgt* RKQQC race.

**Table S13 (see Additional File 6).** GO term enrichment for clusters generated using Pgt genes expressed in the leaves inoculated with the *Pgt* RKQQC race.

**Table S14 (see Additional File 6).** Enrichment of effector encoding genes in gene clusters generated using the RKQQC dataset.

**Table S15 (see Additional File 6).** GO term enrichment for clusters generated using wheat genes expressed in the leaves inoculated with the *Pgt* MCCFC race.

**Table S16 (see Additional File 6).** GO term enrichment for clusters generated using Pgt genes expressed in the leaves inoculated with the *Pgt* MCCFC race.

**Table S17 (see Additional File 6).** Enrichment of effector encoding genes in gene clusters generated using the MCCFC dataset.

**Table S18 (see Additional File 7).** Edges of RKQQC-specific GCN.

**Table S19 (see Additional File 7).** Edges of MCCFC-specific GCN.

**Table S20:** Summary of BLAST2GO enrichment analysis of over represented wheat gene ontology terms in the three network edge conservation groups: MCCFC-specific, RKQQC-specific and conserved.

| **MCCFC-specific wheat network** | | | | | | | | | |
| --- | --- | --- | --- | --- | --- | --- | --- | --- | --- |
| **GO-ID** | **Term** | **GO category (F-Molecular Function, P - Biological Process, C - Cellular Component)** | | **FDR** | **P-Value** | **Test group** | | **Reference group** | |
|  |  |  |  |  |  | **Genes in GO** | **Non GO genes** | **Genes**  **in GO** | **Non GO genes** |
| GO:0016798 | hydrolase activity acting on glycosyl bonds | F | | 1.92E-08 | 2.08E-11 | 39 | 569 | 682 | 38010 |
| GO:0004364 | glutathione transferase activity | F | | 3.94E-07 | 7.81E-10 | 8 | 11 | 713 | 38568 |
| GO:0016639 | oxidoreductase activity acting on the CH-NH2 group of donors NAD or NADP as acceptor | F | | 1.19E-06 | 2.99E-09 | 6 | 3 | 715 | 38576 |
| GO:0004553 | hydrolase activity hydrolyzing O-glycosyl compounds | F | | 1.90E-06 | 5.14E-09 | 30 | 441 | 691 | 38138 |
| GO:0005975 | carbohydrate metabolic process | P | | 1.63E-05 | 6.77E-08 | 52 | 1196 | 669 | 37383 |
| GO:0015930 | glutamate synthase activity | F | | 2.35E-05 | 1.10E-07 | 5 | 3 | 716 | 38576 |
| GO:0016638 | oxidoreductase activity acting on the CH-NH2 group of donors | F | | 2.47E-05 | 1.29E-07 | 7 | 16 | 714 | 38563 |
| GO:0016787 | hydrolase activity | F | | 1.40E-04 | 8.85E-07 | 142 | 5039 | 579 | 33540 |
| GO:0005576 | extracellular region | C | | 5.19E-04 | 3.55E-06 | 21 | 336 | 700 | 38243 |
| GO:0003824 | catalytic activity | F | | 1.31E-03 | 1.23E-05 | 431 | 19886 | 290 | 18693 |
| GO:0016829 | lyase activity | F | | 1.31E-03 | 1.26E-05 | 34 | 772 | 687 | 37807 |
| GO:0016841 | ammonia-lyase activity | F | | 2.25E-03 | 2.42E-05 | 9 | 75 | 712 | 38504 |
| GO:0045181 | glutamate synthase activity NAD(P)H as acceptor | F | | 2.25E-03 | 2.43E-05 | 3 | 1 | 718 | 38578 |
| GO:0000325 | plant-type vacuole | C | | 3.25E-03 | 3.92E-05 | 11 | 123 | 710 | 38456 |
| GO:0003993 | acid phosphatase activity | F | | 3.49E-03 | 4.28E-05 | 8 | 62 | 713 | 38517 |
| GO:0016762 | xyloglucan:xyloglucosyl transferase activity | F | | 5.25E-03 | 7.04E-05 | 4 | 9 | 717 | 38570 |
| GO:1901605 | alpha-amino acid metabolic process | P | | 5.25E-03 | 7.10E-05 | 22 | 448 | 699 | 38131 |
| GO:0009751 | response to salicylic acid | P | | 5.87E-03 | 8.03E-05 | 5 | 20 | 716 | 38559 |
| GO:0016840 | carbon-nitrogen lyase activity | F | | 7.27E-03 | 1.04E-04 | 9 | 92 | 712 | 38487 |
| GO:0016757 | transferase activity transferring glycosyl groups | F | | 1.45E-02 | 2.18E-04 | 31 | 802 | 690 | 37777 |
| GO:0006308 | DNA catabolic process | P | | 2.01E-02 | 3.21E-04 | 3 | 5 | 718 | 38574 |
| GO:0006537 | glutamate biosynthetic process | P | | 2.03E-02 | 3.36E-04 | 2 | 0 | 719 | 38579 |
| GO:0004353 | glutamate dehydrogenase [NAD(P)+] activity | F | | 2.03E-02 | 3.36E-04 | 2 | 0 | 719 | 38579 |
| GO:0000373 | Group II intron splicing | P | | 2.03E-02 | 3.36E-04 | 2 | 0 | 719 | 38579 |
| GO:0043169 | cation binding | F | | 2.28E-02 | 3.91E-04 | 94 | 3474 | 627 | 35105 |
| GO:0009658 | chloroplast organization | P | | 2.69E-02 | 4.89E-04 | 9 | 115 | 712 | 38464 |
| GO:0006536 | glutamate metabolic process | P | | 2.83E-02 | 5.24E-04 | 4 | 17 | 717 | 38562 |
| GO:0018871 | 1-aminocyclopropane-1-carboxylate metabolic process | P | | 3.44E-02 | 6.70E-04 | 3 | 7 | 718 | 38572 |
| GO:0042218 | 1-aminocyclopropane-1-carboxylate biosynthetic process | P | | 3.44E-02 | 6.70E-04 | 3 | 7 | 718 | 38572 |
| GO:0016847 | 1-aminocyclopropane-1-carboxylate synthase activity | F | | 3.44E-02 | 6.70E-04 | 3 | 7 | 718 | 38572 |
| GO:0072330 | monocarboxylic acid biosynthetic process | P | | 4.36E-02 | 8.87E-04 | 13 | 240 | 708 | 38339 |
| GO:0004352 | glutamate dehydrogenase (NAD+) activity | F | | 4.73E-02 | 9.96E-04 | 2 | 1 | 719 | 38578 |
| **RKQQC-specific wheat networks** | | | | | | | | | |
| **GO-ID** | **Term** | | **Category** | **FDR** | **P-Value** | **Test group** | | **Reference group** | |
|  |  |  |  |  |  | **Genes in GO** | **Non GO genes** | **Genes**  **in GO** | **Non GO genes** |
| GO:0009765 | photosynthesis light harvesting | | P | 9.68E-12 | 3.49E-15 | 20 | 87 | 671 | 38522 |
| GO:0004364 | glutathione transferase activity | | F | 9.55E-09 | 1.20E-11 | 9 | 10 | 682 | 38599 |
| GO:0009295 | nucleoid | | C | 1.61E-08 | 2.61E-11 | 10 | 18 | 681 | 38591 |
| GO:0009330 | DNA topoisomerase complex (ATP-hydrolyzing) | | C | 8.60E-07 | 2.32E-09 | 6 | 3 | 685 | 38606 |
| GO:0016639 | oxidoreductase activity acting on the CH-NH2 group of donors NAD or NADP as acceptor | | F | 8.60E-07 | 2.32E-09 | 6 | 3 | 685 | 38606 |
| GO:0016798 | hydrolase activity acting on glycosyl bonds | | F | 4.62E-06 | 1.58E-08 | 33 | 575 | 658 | 38034 |
| GO:0015930 | glutamate synthase activity | | F | 2.35E-05 | 8.88E-08 | 5 | 3 | 686 | 38606 |
| GO:0016638 | oxidoreductase activity acting on the CH-NH2 group of donors | | F | 2.42E-05 | 9.67E-08 | 7 | 16 | 684 | 38593 |
| GO:0003918 | DNA topoisomerase type II (ATP-hydrolyzing) activity | | F | 1.55E-04 | 9.08E-07 | 6 | 14 | 685 | 38595 |
| GO:0061505 | DNA topoisomerase II activity | | F | 1.55E-04 | 9.08E-07 | 6 | 14 | 685 | 38595 |
| GO:0004553 | hydrolase activity hydrolyzing O-glycosyl compounds | | F | 2.06E-04 | 1.30E-06 | 25 | 446 | 666 | 38163 |
| GO:0006772 | thiamine metabolic process | | P | 9.41E-04 | 6.27E-06 | 4 | 4 | 687 | 38605 |
| GO:0000229 | cytoplasmic chromosome | | C | 1.37E-03 | 1.11E-05 | 4 | 5 | 687 | 38604 |
| GO:0009508 | plastid chromosome | | C | 1.37E-03 | 1.11E-05 | 4 | 5 | 687 | 38604 |
| GO:0042646 | plastid nucleoid | | C | 1.37E-03 | 1.11E-05 | 4 | 5 | 687 | 38604 |
| GO:0016787 | hydrolase activity | | F | 1.38E-03 | 1.14E-05 | 132 | 5049 | 559 | 33560 |
| GO:0009658 | chloroplast organization | | P | 1.46E-03 | 1.29E-05 | 11 | 113 | 680 | 38496 |
| GO:1901605 | alpha-amino acid metabolic process | | P | 1.46E-03 | 1.27E-05 | 23 | 447 | 668 | 38162 |
| GO:0016765 | transferase activity transferring alkyl or aryl (other than methyl) groups | | F | 1.82E-03 | 1.74E-05 | 11 | 117 | 680 | 38492 |
| GO:0016841 | ammonia-lyase activity | | F | 1.82E-03 | 1.74E-05 | 9 | 75 | 682 | 38534 |
| GO:0045181 | glutamate synthase activity NAD(P)H as acceptor | | F | 2.20E-03 | 2.14E-05 | 3 | 1 | 688 | 38608 |
| GO:0042723 | thiamine-containing compound metabolic process | | P | 2.83E-03 | 2.83E-05 | 4 | 7 | 687 | 38602 |
| GO:0006265 | DNA topological change | | P | 2.91E-03 | 3.04E-05 | 6 | 29 | 685 | 38580 |
| GO:0004067 | asparaginase activity | | F | 4.37E-03 | 5.27E-05 | 3 | 2 | 688 | 38607 |
| GO:0009228 | thiamine biosynthetic process | | P | 4.37E-03 | 5.27E-05 | 3 | 2 | 688 | 38607 |
| GO:0042724 | thiamine-containing compound biosynthetic process | | P | 4.37E-03 | 5.27E-05 | 3 | 2 | 688 | 38607 |
| GO:0009751 | response to salicylic acid | | P | 5.29E-03 | 6.57E-05 | 5 | 20 | 686 | 38589 |
| GO:0016840 | carbon-nitrogen lyase activity | | F | 5.91E-03 | 7.56E-05 | 9 | 92 | 682 | 38517 |
| GO:0003916 | DNA topoisomerase activity | | F | 5.94E-03 | 7.70E-05 | 6 | 35 | 685 | 38574 |
| GO:0004419 | hydroxymethylglutaryl-CoA lyase activity | | F | 7.50E-03 | 1.04E-04 | 3 | 3 | 688 | 38606 |
| GO:0009532 | plastid stroma | | C | 7.93E-03 | 1.11E-04 | 14 | 228 | 677 | 38381 |
| GO:0000325 | plant-type vacuole | | C | 9.38E-03 | 1.37E-04 | 10 | 124 | 681 | 38485 |
| GO:0006308 | DNA catabolic process | | P | 1.88E-02 | 2.84E-04 | 3 | 5 | 688 | 38604 |
| GO:0043562 | cellular response to nitrogen levels | | P | 1.88E-02 | 2.84E-04 | 3 | 5 | 688 | 38604 |
| GO:0009646 | response to absence of light | | P | 1.94E-02 | 2.98E-04 | 4 | 15 | 687 | 38594 |
| GO:0004353 | glutamate dehydrogenase [NAD(P)+] activity | | F | 1.97E-02 | 3.09E-04 | 2 | 0 | 689 | 38609 |
| GO:0006537 | glutamate biosynthetic process | | P | 1.97E-02 | 3.09E-04 | 2 | 0 | 689 | 38609 |
| GO:0009642 | response to light intensity | | P | 2.19E-02 | 3.47E-04 | 5 | 30 | 686 | 38579 |
| GO:0006536 | glutamate metabolic process | | P | 2.76E-02 | 4.47E-04 | 4 | 17 | 687 | 38592 |
| GO:0006767 | water-soluble vitamin metabolic process | | P | 2.76E-02 | 4.53E-04 | 5 | 32 | 686 | 38577 |
| GO:0006766 | vitamin metabolic process | | P | 3.10E-02 | 5.14E-04 | 5 | 33 | 686 | 38576 |
| GO:0008094 | DNA-dependent ATPase activity | | F | 3.40E-02 | 5.69E-04 | 7 | 74 | 684 | 38535 |
| GO:0009657 | plastid organization | | P | 3.77E-02 | 6.39E-04 | 11 | 181 | 680 | 38428 |
| **Conserved wheat** | | | | | | | | | |
| **GO-ID** | **Term** | | **Category** | **FDR** | **P-Value** | **Test group** | | **Reference group** | |
|  |  |  |  |  |  | **Genes in GO** | **Non GO genes** | **Genes**  **in GO** | **Non GO genes** |
| GO:0016639 | oxidoreductase activity acting on the CH-NH2 group of donors NAD or NADP as acceptor | | F | 1.05E-07 | 5.67E-11 | 6 | 3 | 366 | 38925 |
| GO:0016798 | hydrolase activity acting on glycosyl bonds | | F | 3.29E-07 | 2.37E-10 | 26 | 582 | 346 | 38346 |
| GO:0016638 | oxidoreductase activity acting on the CH-NH2 group of donors | | F | 1.18E-06 | 1.38E-09 | 7 | 16 | 365 | 38912 |
| GO:0015930 | glutamate synthase activity | | F | 2.81E-06 | 4.05E-09 | 5 | 3 | 367 | 38925 |
| GO:0016841 | ammonia-lyase activity | | F | 4.66E-05 | 1.09E-07 | 9 | 75 | 363 | 38853 |
| GO:0016840 | carbon-nitrogen lyase activity | | F | 1.57E-04 | 5.38E-07 | 9 | 92 | 363 | 38836 |
| GO:0000325 | plant-type vacuole | | C | 1.83E-04 | 6.60E-07 | 10 | 124 | 362 | 38804 |
| GO:1901605 | alpha-amino acid metabolic process | | P | 1.86E-04 | 7.20E-07 | 18 | 452 | 354 | 38476 |
| GO:0004553 | hydrolase activity hydrolyzing O-glycosyl compounds | | F | 1.86E-04 | 7.42E-07 | 18 | 453 | 354 | 38475 |
| GO:0004364 | glutathione transferase activity | | F | 1.86E-04 | 7.71E-07 | 5 | 14 | 367 | 38914 |
| GO:0016787 | hydrolase activity | | F | 3.02E-04 | 1.40E-06 | 83 | 5098 | 289 | 33830 |
| GO:0045181 | glutamate synthase activity NAD(P)H as acceptor | | F | 6.40E-04 | 3.34E-06 | 3 | 1 | 369 | 38927 |
| GO:0006536 | glutamate metabolic process | | P | 5.64E-03 | 4.16E-05 | 4 | 17 | 368 | 38911 |
| GO:0006308 | DNA catabolic process | | P | 5.99E-03 | 4.55E-05 | 3 | 5 | 369 | 38923 |
| GO:0009064 | glutamine family amino acid metabolic process | | P | 9.69E-03 | 8.03E-05 | 6 | 69 | 366 | 38859 |
| GO:0003824 | catalytic activity | | F | 1.01E-02 | 8.84E-05 | 230 | 20087 | 142 | 18841 |
| GO:0006537 | glutamate biosynthetic process | | P | 1.01E-02 | 8.94E-05 | 2 | 0 | 370 | 38928 |
| GO:0004353 | glutamate dehydrogenase [NAD(P)+] activity | | F | 1.01E-02 | 8.94E-05 | 2 | 0 | 370 | 38928 |
| GO:0019752 | carboxylic acid metabolic process | | P | 1.66E-02 | 1.49E-04 | 29 | 1416 | 343 | 37512 |
| GO:0006520 | cellular amino acid metabolic process | | P | 2.21E-02 | 2.23E-04 | 20 | 835 | 352 | 38093 |
| GO:0009698 | phenylpropanoid metabolic process | | P | 2.24E-02 | 2.34E-04 | 6 | 85 | 366 | 38843 |
| GO:0043436 | oxoacid metabolic process | | P | 2.39E-02 | 2.64E-04 | 29 | 1443 | 343 | 37485 |
| GO:0004352 | glutamate dehydrogenase (NAD+) activity | | F | 2.39E-02 | 2.66E-04 | 2 | 1 | 370 | 38927 |
| GO:0006082 | organic acid metabolic process | | P | 2.39E-02 | 2.67E-04 | 29 | 1445 | 343 | 37483 |
| GO:1901606 | alpha-amino acid catabolic process | | P | 2.54E-02 | 2.88E-04 | 7 | 126 | 365 | 38802 |
| GO:0009063 | cellular amino acid catabolic process | | P | 2.58E-02 | 3.02E-04 | 7 | 127 | 365 | 38801 |
| GO:0009084 | glutamine family amino acid biosynthetic process | | P | 3.67E-02 | 5.00E-04 | 4 | 35 | 368 | 38893 |
| GO:0004604 | phosphoadenylyl-sulfate reductase (thioredoxin) activity | | F | 3.67E-02 | 5.29E-04 | 2 | 2 | 370 | 38926 |
| GO:0047395 | glycerophosphoinositol glycerophosphodiesterase activity | | F | 3.67E-02 | 5.29E-04 | 2 | 2 | 370 | 38926 |
| GO:0019419 | sulfate reduction | | P | 3.67E-02 | 5.29E-04 | 2 | 2 | 370 | 38926 |
| GO:0019379 | sulfate assimilation phosphoadenylyl sulfate reduction by phosphoadenylyl-sulfate reductase (thioredoxin) | | P | 3.67E-02 | 5.29E-04 | 2 | 2 | 370 | 38926 |
| GO:0009072 | aromatic amino acid family metabolic process | | P | 3.67E-02 | 5.49E-04 | 7 | 141 | 365 | 38787 |
| GO:0006559 | L-phenylalanine catabolic process | | P | 3.77E-02 | 5.77E-04 | 5 | 66 | 367 | 38862 |
| GO:1902222 | erythrose 4-phosphate/phosphoenolpyruvate family amino acid catabolic process | | P | 3.77E-02 | 5.77E-04 | 5 | 66 | 367 | 38862 |
| GO:0005576 | extracellular region | | C | 4.25E-02 | 6.65E-04 | 11 | 346 | 361 | 38582 |
| GO:0005773 | vacuole | | C | 4.35E-02 | 6.92E-04 | 18 | 780 | 354 | 38148 |
| GO:0009074 | aromatic amino acid family catabolic process | | P | 4.35E-02 | 6.98E-04 | 5 | 69 | 367 | 38859 |
| GO:0022804 | active transmembrane transporter activity | | F | 4.39E-02 | 7.12E-04 | 20 | 901 | 352 | 38027 |
| GO:0006558 | L-phenylalanine metabolic process | | P | 4.48E-02 | 7.42E-04 | 5 | 70 | 367 | 38858 |
| GO:1902221 | erythrose 4-phosphate/phosphoenolpyruvate family amino acid metabolic process | | P | 4.48E-02 | 7.42E-04 | 5 | 70 | 367 | 38858 |
| GO:0005774 | vacuolar membrane | | C | 4.56E-02 | 7.63E-04 | 14 | 529 | 358 | 38399 |
| GO:0044437 | vacuolar part | | C | 4.65E-02 | 7.90E-04 | 14 | 531 | 358 | 38397 |
| GO:0006040 | amino sugar metabolic process | | P | 4.65E-02 | 7.95E-04 | 4 | 40 | 368 | 38888 |
| GO:0009973 | adenylyl-sulfate reductase activity | | F | 4.92E-02 | 8.77E-04 | 2 | 3 | 370 | 38925 |

**Table S21:** Summary of metadata for the combined GO sub-networks (*see Figure 2 for details on inter-isolate genomic conservation groups of effector candidates).

| **GO terms (redundant terms are combined)** | **Number GO annotated genes** | **Number conserved GO annotated genes** | **Total edges in combined sub-network** | **Edges conserved between sub-networks** | **Number wheat genes** | **Wheat genes conserved between sub-networks** | **Number effector candidates** | **Effector candidates conserved between sub-networks** | ***Effector candidates in genomic cons group1** | ***Effector candidates in genomic cons group2** | ***Effector candidates in genomic cons group3** |
| --- | --- | --- | --- | --- | --- | --- | --- | --- | --- | --- | --- |
| GO_0000229, GO_0042646, GO_0009508 | 3 | 1 | 66 | 0  (0%) | 28 | 1  (3.6%) | 15 | 0  (0%) | 1 | 5 | 9 |
| GO_0009295 | 9 | 3 | 97 | 0  (0%) | 117 | 6  (5.1%) | 23 | 1  (4.3%) | 2 | 8 | 13 |
| GO_0009765 | 20 | 1 | 1,068 | 0  (0%) | 77 | 1  (1.3%) | 1 | 0  (0%) | 0 | 0 | 1 |
| GO_0004067 | 3 | 2 | 4,944 | 1  (0%) | 164 | 4  (2.4%) | 7 | 0  (0%) | 0 | 1 | 6 |
| GO_0008094, GO_0003918, GO_0006265, GO_0009330, GO_0003916, GO_0061505 | 7 | 2 | 1200 | 1  (0.1%) | 98 | 5  (5.1%) | 9 | 0  (0%) | 1 | 4 | 4 |
| GO_0016757 | 30 | 19 | 765 | 4  (0.5%) | 1167 | 344  (29.5%) | 93 | 11  (11.8%) | 11 | 33 | 49 |
| GO_0009642 | 5 | 3 | 5937 | 32  (0.5%) | 262 | 23  (8.8%) | 39 | 0  (0%) | 6 | 11 | 22 |
| GO_0009646 | 4 | 2 | 5676 | 28  (0.5%) | 250 | 20  (8%) | 38 | 0  (0%) | 6 | 10 | 22 |
| GO_0009658, GO_0009657 | 14 | 6 | 11859 | 86  (0.7%) | 424 | 45  (10.6%) | 57 | 1  (1.8%) | 7 | 16 | 34 |
| GO_0003993 | 8 | 5 | 6965 | 60  (0.9%) | 189 | 15  (7.9%) | 5 | 0  (0%) | 1 | 1 | 3 |
| GO_0009532 | 15 | 7 | 254 | 3  (1.2%) | 688 | 243  (35.3%) | 49 | 3  (6.1%) | 8 | 11 | 30 |
| GO_0016762 | 4 | 1 | 8491 | 139  (1.6%) | 268 | 24  (9%) | 25 | 1  (4%) | 4 | 6 | 15 |
| GO_0006308 | 3 | 3 | 7436 | 162  (2.2%) | 215 | 32  (14.9%) | 25 | 2  (8%) | 1 | 10 | 14 |
| GO_0003824 | 525 | 305 | 4009 | 93  (2.3%) | 2097 | 1161  (55.4%) | 236 | 69  (29.2%) | 13 | 91 | 132 |
| GO_0006766, GO_0006767, GO_0042723, GO_0006772 | 5 | 1 | 129 | 3  (2.3%) | 22 | 3  (13.6%) | 2 | 0  (0%) | 1 | 0 | 1 |
| GO_0005975 | 51 | 27 | 1135 | 28  (2.5%) | 1402 | 574  (40.9%) | 121 | 26  (21.5%) | 9 | 42 | 70 |
| GO_0009751 | 5 | 5 | 13157 | 350  (2.7%) | 336 | 53  (15.8%) | 27 | 2  (7.4%) | 0 | 11 | 16 |
| GO_0006040 | 6 | 4 | 15696 | 479  (3.1%) | 423 | 61  (14.4%) | 32 | 0  (0%) | 2 | 12 | 18 |
| GO_0016847, GO_0018871, GO_0042218 | 3 | 2 | 4333 | 154  (3.6%) | 149 | 27  (18.1%) | 23 | 0  (0%) | 0 | 9 | 14 |
| GO_0005576 | 24 | 13 | 44850 | 2884  (6.4%) | 858 | 193  (22.5%) | 64 | 4  (6.2%) | 7 | 20 | 37 |
| GO_0022804 | 32 | 23 | 62110 | 5301  (8.5%) | 1059 | 364  (34.4%) | 96 | 9  (9.4%) | 11 | 36 | 49 |
| GO_0043562 | 3 | 2 | 10597 | 930  (8.8%) | 314 | 47  (15%) | 13 | 0  (0%) | 2 | 4 | 7 |
| GO_0005773 | 29 | 21 | 74101 | 6779  (9.1%) | 1120 | 395  (35.3%) | 101 | 19  (18.8%) | 9 | 37 | 55 |
| GO_0044437, GO_0005774 | 17 | 15 | 62769 | 6082  (9.7%) | 1006 | 331  (32.9%) | 78 | 11  (14.1%) | 9 | 24 | 45 |
| GO_0043169 | 94 | 57 | 106651 | 12756  (12%) | 1577 | 714  (45.3%) | 149 | 40  (26.8%) | 10 | 62 | 77 |
| GO_0006536 | 4 | 4 | 19465 | 2462  (12.6%) | 354 | 109  (30.8%) | 5 | 0  (0%) | 0 | 1 | 4 |
| GO_0000325 | 11 | 10 | 31251 | 3937  (12.6%) | 626 | 193  (30.8%) | 33 | 3  (9.1%) | 6 | 6 | 21 |
| GO_0016787 | 176 | 99 | 120245 | 15365  (12.8%) | 1832 | 932  (50.9%) | 205 | 50  (24.4%) | 13 | 79 | 113 |
| GO_0004353, GO_0004352 | 2 | 2 | 16613 | 2159  (13%) | 308 | 98  (31.8%) | 4 | 0  (0%) | 0 | 2 | 2 |
| GO_0019752, GO_0043436, GO_0006082 | 50 | 34 | 81688 | 11089  (13.6%) | 1149 | 462  (40.2%) | 108 | 6  (5.6%) | 6 | 41 | 61 |
| GO_0016638 | 7 | 7 | 19814 | 2721  (13.7%) | 392 | 125  (31.9%) | 9 | 0  (0%) | 0 | 3 | 6 |
| GO_0016639 | 6 | 6 | 19729 | 2712  (13.7%) | 390 | 124  (31.8%) | 9 | 0  (0%) | 0 | 3 | 6 |
| GO_0072330 | 13 | 8 | 43743 | 6197  (14.2%) | 673 | 224  (33.3%) | 60 | 2  (3.3%) | 1 | 24 | 35 |
| GO_0004553 | 35 | 20 | 81362 | 11865  (14.6%) | 1285 | 485  (37.7%) | 91 | 13  (14.3%) | 7 | 30 | 54 |
| GO_0016798 | 44 | 28 | 87476 | 12977  (14.8%) | 1348 | 549  (40.7%) | 93 | 15  (16.1%) | 7 | 32 | 54 |
| GO_0006520 | 31 | 22 | 57847 | 9788  (16.9%) | 851 | 356  (41.8%) | 54 | 1  (1.9%) | 1 | 21 | 32 |
| GO_1901605 | 25 | 20 | 56995 | 9774  (17.1%) | 839 | 353  (42.1%) | 52 | 1  (1.9%) | 1 | 20 | 31 |
| GO_0045181 | 3 | 3 | 6738 | 1191  (17.7%) | 178 | 56  (31.5%) | 2 | 0  (0%) | 0 | 0 | 2 |
| GO_0015930 | 5 | 5 | 8791 | 1605  (18.3%) | 232 | 73  (31.5%) | 5 | 0  (0%) | 0 | 1 | 4 |
| GO_0006537 | 2 | 2 | 7255 | 1381  (19%) | 174 | 62  (35.6%) | 2 | 0  (0%) | 0 | 0 | 2 |
| GO_0009064 | 6 | 6 | 31328 | 5979  (19.1%) | 527 | 203  (38.5%) | 10 | 0  (0%) | 1 | 2 | 7 |
| GO_0009973, GO_0004604, GO_0019379, GO_0019419 | 2 | 2 | 17817 | 3453  (19.4%) | 349 | 129  (37%) | 1 | 0  (0%) | 1 | 0 | 0 |
| GO_0016829 | 39 | 22 | 51157 | 10224  (20%) | 705 | 326  (46.2%) | 32 | 0  (0%) | 2 | 11 | 19 |
| GO_0016765, GO_0004364 | 11 | 8 | 34913 | 7235  (20.7%) | 574 | 223  (38.9%) | 16 | 0  (0%) | 1 | 6 | 9 |
| GO_0047395 | 2 | 2 | 26855 | 5802  (21.6%) | 427 | 195  (45.7%) | 1 | 0  (0%) | 0 | 0 | 1 |
| GO_0009698 | 8 | 6 | 29144 | 6443  (22.1%) | 473 | 197  (41.6%) | 2 | 0  (0%) | 0 | 1 | 1 |
| GO_0009084 | 4 | 4 | 22843 | 5096  (22.3%) | 418 | 158  (37.8%) | 7 | 0  (0%) | 1 | 1 | 5 |
| GO_1901606, GO_0009063 | 7 | 7 | 32496 | 7925  (24.4%) | 464 | 234  (50.4%) | 3 | 0  (0%) | 0 | 1 | 2 |
| GO_0009072 | 9 | 8 | 30163 | 7460  (24.7%) | 453 | 215  (47.5%) | 3 | 0  (0%) | 0 | 0 | 3 |
| GO_0006559, GO_1902222, GO_1902221, GO_0009074, GO_0006558 | 5 | 5 | 26760 | 7036  (26.3%) | 384 | 197  (51.3%) | 0 | 0  (0%) | 0 | 0 | 0 |
| GO_0016840, GO_0016841 | 9 | 9 | 27317 | 7305  (26.7%) | 390 | 208  (53.3%) | 1 | 0  (0%) | 0 | 0 | 1 |

**Table S22 (see Additional File 8).** MYB-responsive elements in the promoter regions of the SA-associated subnetwork (GO: 0009751).
